# Fast and accurate population admixture inference from genotype data from a few microsatellites to millions of SNPs

**DOI:** 10.1038/s41437-022-00535-z

**Published:** 2022-05-04

**Authors:** Jinliang Wang

**Affiliations:** Institute of Zoology, Zoological Society of London, London, NW1 4RY UK

**Keywords:** Population genetics, Evolutionary ecology

## Abstract

Model-based (likelihood and Bayesian) and non-model-based (PCA and *K*-means clustering) methods were developed to identify populations and assign individuals to the identified populations using marker genotype data. Model-based methods are favoured because they are based on a probabilistic model of population genetics with biologically meaningful parameters and thus produce results that are easily interpretable and applicable. Furthermore, they often yield more accurate structure inferences than non-model-based methods. However, current model-based methods either are computationally demanding and thus applicable to small problems only or use simplified admixture models that could yield inaccurate results in difficult situations such as unbalanced sampling. In this study, I propose new likelihood methods for fast and accurate population admixture inference using genotype data from a few multiallelic microsatellites to millions of diallelic SNPs. The methods conduct first a clustering analysis of coarse-grained population structure by using the mixture model and the simulated annealing algorithm, and then an admixture analysis of fine-grained population structure by using the clustering results as a starting point in an expectation maximisation algorithm. Extensive analyses of both simulated and empirical data show that the new methods compare favourably with existing methods in both accuracy and running speed. They can analyse small datasets with just a few multiallelic microsatellites but can also handle in parallel terabytes of data with millions of markers and millions of individuals. In difficult situations such as many and/or lowly differentiated populations, unbalanced or very small samples of individuals, the new methods are substantially more accurate than other methods.

## Introduction

Inferring the genetic structure of a population at family and subpopulation levels from a sample of multilocus genotypes is important for understanding the acting evolutionary forces and population demography, for managing populations in conservation (Crandall et al. 2000), and for controlling the stratification in genome wide association studies (GWAS) of complex traits such as inheritable diseases (Francioli et al. 2014). We can use genotype data in reconstructing the pedigree that underlies the data (e.g., Wang and Santure 2009), and in detecting subdivision, identifying subpopulations and assigning sampled individuals to the inferred populations (e.g., Pritchard et al. 2000). Indeed, pedigree and subdivision are both genetic structures, the former being a structure at a (finer) family level and the latter being a structure at a (coarser) subpopulation level. In reality, however, the two structures can be difficult to distinguish because a family can be regarded as a subpopulation and a subpopulation can be regarded as an extended family.

Many methods have been proposed to estimate population-level structure since the seminal work of Pritchard et al. (2000). They can be classified into two broad categories, model-based and non-model-based methods. The former is in a probabilistic framework built from a population genetics model. When implemented in a Bayesian approach, it has a pivotal function of the probability of genotype data conditional on population parameters such as individual ancestry and allele frequencies in inferred populations. Sampling (by Markov Chain Monte Carlo, MCMC) from the posterior distributions of the parameters given the data and some suitably selected priors (e.g., Pritchard et al. 2000; Raj et al. 2014; Gopalan et al. 2016) yields Bayesian estimates of the parameters. When implemented in a likelihood approach, it has a likelihood function of the parameters which are estimated by maximising the function (e.g., Tang et al. 2005; Alexander et al. 2009). Non-model-based methods are not based on a probabilistic population genetics model, but on more generic statistical approaches such as principal component analysis (PCA, Patterson et al. 2006; Price et al. 2006) and *K*-means clustering analysis (Jombart et al. 2010). The former projects high-dimensional genotype data into a few orthogonal variables, called principal components (PCs), which (hopefully) summarize the data well and make it possible for the clustering of sampled individuals and for the visualisation of the clustering. The latter classifies individuals directly into a predefined number of clusters by minimising the genetic differences within a cluster and maximising the genetic differences between clusters. Compared with model-based methods, non-model methods make much fewer assumptions and are thus more robust, computationally much faster, and applicable to larger datasets. However, because of the lack of a solid population genetics model, non-model methods give results that are much harder to interpret and apply. PCA results can also be confounded by demographic factors or irregular sampling designs (Novembre and Stephens 2008; McVean 2009).

The first and most popular Bayesian method was proposed and implemented in the software STRUCTURE by Pritchard et al. (2000). Further work extended and improved the method substantially (Falush et al. 2003; 2007; Hubisz et al. 2009). Many similar methods were also developed and applied (e.g., Dawson and Belkhir 2001; Corander et al. 2003; Gao et al. 2007; Huelsenbeck and Andolfatto 2007). However, STRUCTURE remains unequivocally the most popular because of its elaborated models (e.g., correlated and uncorrelated allele frequency models, Falush et al. 2003), robustness to marker genotyping problems (e.g., null or recessive alleles, Falush et al. 2007), and ease of use (e.g., Windows graphical user interface). STRUCTURE works well for small datasets. However, it becomes infeasible computationally to apply to a large sample of genomic markers or individuals, or to an analysis with many populations. With the rapid developments in sequencing technology, data of genome wide SNPs in thousands (Leslie et al. 2015) or hundreds of thousands (Bryc et al. 2015) of individuals are increasingly collected to study the fine-scale genetic structure or to hunt for disease genes in genome wide association studies (GWAS). STRUCTURE was not designed for and thus incapable of handling genotype data at this large scale, in gigabytes or even terabytes (Gopalan et al. 2016; Bose et al. 2019).

Since the development of STRUCTURE, two noticeable advances have been made to use large-scale genomic data in model-based admixture analysis. One was initiated by Tang et al. (2005), who proposed a likelihood method of the admixture model and developed an expectation maximisation (EM) algorithm for maximum likelihood estimation of both individual admixture proportions (ancestries) and population allele frequencies. Alexander et al. (2009) improved the computational efficiency greatly by introducing into the EM algorithm a fast block relaxation scheme using sequential quadratic programming for block updates. The software ADMIXTURE, which implements the algorithm, turns out to be a great success, being used routinely in analysing large-scale genomic data. The same likelihood function can also be solved by an even faster algorithm, sparse nonnegative matrix factorisation (sNMF) and least-squares optimisation, developed by Frichot et al. (2014). Another development was to use variational Bayesian inference method to approximate the relevant posterior distributions as an optimisation problem (Raj et al. 2014; Gopalan et al. 2016). Implementing the method in software fastSTRUCTURE, Raj et al. (2014) showed that it could handle large-scale genomic data as fast as and as accurate as ADMIXTURE. Gopalan et al. (2016) implemented the method in software TeraStructure which can deal with an unprecedented amount of genotype data (e.g., 1 million individuals genotyped at 1 million loci). Both fastSTRUCTURE and TeraStructure run much faster but are much less flexible (e.g., unable to handle multiallelic or recessive markers) and less accurate than STRUCTURE.

All developments of Pritchard et al.’s probabilistic model of STRUCTURE were either on improving computational efficiency (above), or on extending the model for application to tricky data (e.g., recessive markers, Falush et al. 2007; Hubisz et al. 2009) or non-standard populations (e.g., inbreeding, Gao et al. 2007). Little attention is paid to investigating and improving the convergence of the algorithms and the accuracy of the method in difficult situations such as very small or highly unbalanced samples of individuals, low differentiation, many populations, or a large sample of markers and individuals. This is unfortunate as the admixture model is high-dimensional, containing many variables to estimate jointly. As a result, the model, implemented in either Bayesian or likelihood framework, may have a high risk of non-convergence or converging to a local rather than global optimum. A typical admixture analysis must handle roughly *V* = (*K* − 1)*N* + (*A* − *L*)*K* (independent) variables, where the admixture proportions of *N* individuals and the allele frequencies in *K* source populations are to be inferred from the genotype data at *L* loci with a total number of *A* alleles. Current methods face an increasing risk of non-convergence with an increase in the scale of the problem determined mainly by *V*, as well as an increase in the complexity of population structure. They may not converge even for a small problem (small *V*, say *V* = 76 when *K* = 4, *N* = 12, *A* = 20 and *L* = 10) in difficult situations such as when few individuals are sampled from a source population or vastly different numbers of individuals are sampled from different populations. The EM and sNMF algorithms in the likelihood framework make no effort (except for the suggestion of making multiple replicate runs) in seeking the global rather than local maximum likelihood, just like other generic clustering approaches such as *K*-means method. These, like the MCMC algorithm in the Bayesian framework, may fail to converge in a large-scale admixture analysis.

In this study, I propose a two-step procedure to infer population structure and individual admixture proportions from genotype data. First, I assume a mixture model (i.e., no admixture) to conduct a clustering analysis (i.e., assigning individuals to distinct clusters with each representing a population), using the simulated annealing algorithm with extra care to avoid converging to local maxima of the likelihood. Second, I assume an admixture model to conduct an admixture analysis (i.e., estimating individual admixture proportions), using an EM algorithm and the clustering results of the first step as initial values. I implement the approach in a software package PopCluster runnable on all major computer platforms. I show, by using extensive simulated and empirical data, that PopCluster can handle both small and large datasets, from a few multiallelic microsatellites traditionally analysed by STRUCTURE to large genomic datasets with millions of SNPs usually analysed by ADMIXTURE and PCA. I also show that PopCluster converges well and yields more accurate results than other methods in difficult situations such as a small sample of individuals per population, unbalanced sampling, low differentiation, high admixture, and many populations. Coupled with efficient data encoding and parallel computation using both openMP and MPI (Message Passing Interface), PopCluster is capable of handling large datasets from many gigabytes to terabytes that other model-based methods may fail to run or run much slower.

## Methods

### Overall strategy

An admixture analysis aims to estimate the admixture proportions (or ancestries), **Q**, of each sampled individual in a given number of *K* source populations (Pritchard et al. 2000), and the characteristic allele frequencies, **P**, at each locus of each inferred source population. Even though **Q** is frequently of the primary interest, **P** must be estimated simultaneously because we have genotype data only and **Q** is highly dependent on **P** which actually defines the source populations. For *N* individuals from *K* source populations genotyped at *L* loci with a total number of *A* alleles, the numbers of independent variables in **Q** and **P** are *V*_*Q*_ = (*K* − 1)*N* and *V*_*P*_ = (*A* − *L*)*K*, respectively. The high dimensionality of an admixture analysis, with *V* = *V*_*Q*_ + *V*_*P*_ = (*K* − 1)*N* + (*A* − *L*)*K* variables, not only incurs a large computational burden, but also poses a high risk of non-convergence (to the global maximum) for any algorithm, especially when either **Q** or **P** is expected to be poorly estimated in difficult situations such as a small sample (say, a couple) of individuals from each source population or low differentiation.

I propose a two-step procedure with corresponding algorithms to reduce the risk of non-convergence, to speed up the computation, and to make more accurate inferences of both **Q** and **P**. In the first step, I assume a mixture model (Pritchard et al. 2000; Falush et al. 2003) that individuals in a sample can come from different source populations, but each individual’s genome comes exclusively from a single population. Under this simplified probabilistic model, I conduct a clustering analysis to obtain estimates of both individual memberships and allele frequencies of each cluster by a global maximisation algorithm, simulated annealing, with extra care (details below) of convergence. In the absence of admixture and with sufficient information for complete recovery of population structure, the estimated individual memberships and allele frequencies of the clusters are expected to be equivalent to **Q** (with element *q*_*ik*_ = 1 and *q*_*il*_ = 0 if individual *i* is inferred to be in cluster *k* where *l* ≠ *k*) and **P**, respectively. Otherwise, they are expected to be good approximations of **Q** and **P**, because an admixed individual *i* with the highest ancestral proportion from a population would be expected to be assigned (exclusively) to that population. In the second step, I assume an admixture model (Pritchard et al. 2000; Falush et al. 2003) to refine estimates of **Q** and **P**, using an EM algorithm and the start parameter (**Q** and **P**) values obtained from the clustering analysis. Because the starting values are already close to the truth, the algorithm is fast and has a much-reduced risk of converging to a local maximum than the original EM algorithms (Tang et al. 2005; Alexander et al. 2009).

### Clustering analysis

I assume *N* diploid individuals are sampled from *K* source populations. The origin of a sampled individual from the *K* source populations is unknown, which is the primary interest of structure analysis. However, if it is (partially) known, this information can be used to supervise (help) the clustering analysis of other sampled individuals of unknown origins. Each individual’s genome comes exclusively from one of the *K* unknown source populations (i.e., mixture model, no admixture). I assume each individual is genotyped at *L* loci, with a diploid genotype {*x*_*il*1_, *x*_*il*2_} for individual *i* (=1, 2, …, *N*) at locus *l* (=1, 2, …, *L*). The task of the clustering analysis is to sort the *N* individuals with genotype data **X** = {*x*_*ila*_:*i* = 1, 2, …, *N*; *l* = 1, 2, …, *L*; *a* = 1, 2} into *K* clusters, with each representing a source population. No assumption is made about the evolutionary relationships of the populations, which, when summarized by *F* statistics, are estimated from the same genotype data in both clustering and admixture analyses.

Suppose, in a given clustering configuration **Ω** = {**Ω**_1_, **Ω**_2_, …, **Ω**_*K*_}, cluster *k* (=1, 2, …, *K*), **Ω**_*k*_, contains a set of *N*_*k*_ (with *N*_*k*_ > 0 and $\mathop {\sum}\nolimits_{k = 1}^K {N_k \equiv N}$) individuals, denoted by **Ω**_*k*_ = {*ω*_*k*1_, *ω*_*k*2_, …, *ω*_*k*_*N*_*k*_} where *ω*_*kj*_ is the index of the *j*th individual in cluster *k*. The genotype data of the *N*_*k*_ individuals in cluster *k* is **X**_*k*_ = {*x*_*ila*_: *i* ∈ **Ω**_*k*_; *l* = 1, 2*, …, L; a* = 1, 2}. The log-likelihood of **Ω**_*k*_ is then the log probability of observing **X**_*k*_ given **Ω**_*k*_

1 $${{{\mathcal{L}}}}_k\left( {{{{\mathbf{\Omega }}}}_k} \right) = {{{\mathrm{LogP}}}}\left( {{{{\mathbf{X}}}}_k\left| {{{{\mathbf{\Omega }}}}_k} \right.} \right) = \mathop {\sum}\limits_{l = 1}^L {\mathop {\sum}\limits_{j = 1}^{J_l} {c_{klj}{{{\mathrm{Log}}}}\left( {p_{klj}} \right)} }$$

where *c*_*klj*_ and *p*_*klj*_ are the count of copies and the frequency, respectively, of allele *j* at locus *l* in cluster *k*, and *J*_*l*_ is the number of alleles at locus *l*. Given **Ω**_*k*_, *c*_*klj*_ is counted from genotype data **X**_*k*_, and allele frequency *p*_*klj*_ is estimated by

2 $$p_{klj} = \left( {p_{lj} + c_{klj}} \right)/\mathop {\sum}\limits_{m = 1}^{J_l} {\left( {p_{lm} + c_{klm}} \right)}$$

where *p*_*lj*_ is the frequency of allele *j* at locus *l* in the entire population represented by the *K* clusters. *p*_*lj*_ is calculated by

3 $$p_{lj} = \mathop {\sum}\limits_{k = 1}^K {c_{klj}} /\mathop {\sum}\limits_{m = 1}^{J_l} {\mathop {\sum}\limits_{k = 1}^K {c_{klm}} } = c_{lj}/\mathop {\sum}\limits_{m = 1}^{J_l} {c_{lm}}$$

where $c_{lm} = \mathop {\sum}\nolimits_{k = 1}^K {c_{klm}}$ is the count of allele *m* (=1, 2, …, *J*_*l*_) at locus *l* in the entire sample of individuals.

Under the mixture model above, clusters are only weakly dependent (with the extent of dependency decreasing with an increasing value of *K*) and the total log-likelihood of the clustering configuration, **Ω** = {**Ω**_1_, **Ω**_2_, …, **Ω**_*K*_}, is thus

4 $${{{\mathcal{L}}}}\left( {{{\mathbf{\Omega }}}} \right) = \mathop {\sum}\limits_{k = 1}^K {{{{\mathcal{L}}}}_k\left( {{{{\mathbf{\Omega }}}}_k} \right)} ,$$

where ${{{\mathcal{L}}}}_k\left( {{{{\mathbf{\Omega }}}}_k} \right)$ is calculated by (1).

It is worth noting that allele frequencies, **P**, are modelled as hidden or nuisance variables and are estimated as a by-product of maximising (4) for estimates of **Ω**. Yet, careful modelling of **P** proves important for estimating **Ω**, as the two are highly dependent. Bayesian admixture methods assume allele frequencies **p**_*kl*_ = {*p*_*kl*1_, *p*_*kl*2_, …, $p_{klj_l}$} in a Dirichlet distribution (e.g., Foreman et al. 1997; Rannala and Mountain 1997; Pritchard et al. 2000), ${{{\mathcal{D}}}}\left( {\lambda _1,\lambda _2, \ldots ,\lambda _{J_l}} \right)$. For any population *k*, the uncorrelated (Pritchard et al. 2000) and correlated (Falush et al. 2003) allele frequency model assumes *λ*_*j*_ = 1 and $\lambda_j=p_{ol_j}F_K/(1-F_k)$, respectively, for *j* = 1, 2, …, *J*_*l*_. In the latter model, *p*_0*lj*_ is the frequency of allele *j* at locus *l* in the ancestral population (common to the *K* derived populations), and *F*_*k*_ is the differentiation of population *k* from the ancestral population. In contrast, likelihood admixture methods (e.g., Tang et al. 2005; Alexander et al. 2009; Frichot et al. 2014) and non-model based clustering methods (e.g., *K*-means method, Jombart et al. 2010) do not use any prior, which is equivalent to assuming *p*_*lj*_ ≡ 0 for *j* = 1, 2, …, *J*_*l*_ in Eq. (2). However, properly modelling prior allele frequencies, as carefully considered in Bayesian methods (Pritchard et al. 2000; Falush et al. 2003), becomes important in situations where allele frequencies are not well defined or tricky to estimate, such as when few individuals are sampled from a source population or when rare alleles are present. The frequentist estimator (2) is in spirit similar to the Bayesian correlated allele frequency model (Falush et al. 2003), and leads to accurate results in various situations to be shown in this study. I have also tried alternatives such as *p*_*lj*_ ≡ 1/*J*_*l*_ (which is similar to the uncorrelated allele frequency model of Pritchard et al. 2000) or *p*_*lj*_ ≡ 0 (which is equivalent to the treatment in previous likelihood admixture analysis or non-model based clustering analysis) in replacement of (2), but none works as well as (2) and could yield much less accurate results in difficult situations (below).

### Scaling for unbalanced sampling

Bayesian methods of STRUCTURE’s admixture model assume an individual *i*’s ancestry, **q**_*i*_ = {*q*_*i*1_, *q*_*i*2_, …, *q*_*iK*_}, follows a prior Dirichlet probability distribution ${{{\mathbf{q}}}}_i\sim {{{\mathbf{{{{\mathcal{D}}}}}}}}\left( {\alpha _1,\alpha _2, \ldots ,\alpha _K} \right)$ (Pritchard et al. 2000; Falush et al. 2003). By default, *α*_1_ = *α*_2_ = ··· = *α*_*K*_ = *α*, which essentially assumes that an individual has its ancestry originating from each of the assumed *K* populations at an equal prior probability of 1/*K*. To model unequal sample sizes such that an individual comes from a more intensively sampled population at a higher prior probability, STRUCTURE also has applied an alternative prior, *α*_1_ ≠ *α*_2_ ≠ ··· ≠ *α*_*K*_. It is shown that, when sampling intensity is heavily unbalanced among populations, the default prior could lead to the split of a large cluster and the merge of small clusters, while the alternative prior yields much more accurate results (Wang 2017). These priors have a large impact on admixture analysis; applying the default prior to data of highly unbalanced samples leads to inaccurate **Q** estimates even when many informative markers are used (Wang 2017).

Unfortunately, current non-model based or likelihood-based admixture analysis methods do not utilise these or other priors for handling unbalanced sampling. As a result, they can give inaccurate admixture estimates, just like STRUCTURE under the default ancestry prior model, for data from highly unbalanced sampling. To reduce the cluster split and merge problems, herein I propose the following method to scale the likelihood of a cluster by the size, the number of individual members, of the cluster.

The original log-likelihood of cluster *k*, ${{{\mathcal{L}}}}_k\left( {{{{\mathbf{\Omega }}}}_k} \right)$, is calculated by (1). It is then scaled by the cluster size, *N*_*k*_, as

5 $${{{\mathcal{L}}}}_{Sk}\left( {{{{\mathbf{\Omega }}}}_k} \right) = {{{\mathcal{L}}}}_k\left( {{{{\mathbf{\Omega }}}}_k} \right)/\left( {1 + e^{sN_k/\left( {8N} \right)}} \right),$$

where *s* is the scaling factor taking values 1, 2, 3 for weak, medium and strong scaling, respectively. This scaling scheme encourages large clusters and discourages small clusters. Although (5) is not an analytically derived but an empirical equation and is thus not guaranteed to be optimal, extensive simulations (some shown below) verify that the scaling scheme works very well for data from highly unbalanced sampling, yielding accurate clustering analysis results and thus similarly or more accurate admixture estimates than STRUCTURE under its alternative ancestry model. The most appropriate scaling level (1, 2 or 3) for a particular dataset depends on how unbalanced the sampling is, how much differentiated the populations are, and how much informative the markers are. For example, a low scaling level, *s* = 1, is appropriate when many markers are genotyped for a set of lowly differentiated (low *F*_*ST*_) populations. Usually, we do not know these factors in analysing the data. Therefore, when the data are suspected to be unbalanced in sampling among populations, they are better analysed comparatively with different levels of scaling (0, 1, 2, and 3). When the applied level of scaling is too low, large populations tend to be split and small populations tend to be merged. When the applied level of scaling is too high, small populations tend to be merged among themselves or with a large population. With the help of some internal information such as consistency of replicate runs at the same scaling level and the same *K* value and some external information such as sampling locations in examining the admixture estimates, the appropriate scaling level can be determined.

### Simulated annealing algorithm

A likelihood function with many variables, such as (4), is difficult to maximise for estimates of the variables. Traditional methods, such as derivative based Newton-Raphson algorithm (e.g., Tang et al. 2005) and non-derivative based EM algorithm (Dempster et al. 1977; Tang et al. 2005; Alexander et al. 2009), may converge to a local rather than the global maximum for a large scale problem with ridges and plateaus (Gaffe et al. 1994). Although trying multiple replicate runs with different starting values and choosing the run with the highest likelihood could reduce the risk of landing on a local maximum, a global maximum cannot be guaranteed regardless of the number of runs. The Bayesian approach as implemented in STRUCTURE (Pritchard et al. 2000) has a similar problem, as different replicate runs of the same data with the same parameter and model choices but different random number seeds may yield different admixture estimates and likelihood values (Tang et al. 2005; below).

Simulated annealing (SA) was developed to optimise very large and complex systems (Kirkpatrick et al. 1983). Using the Metropolis algorithm (Metropolis et al. 1953) from statistical mechanics, SA can find the global maximum by searching both downhill and uphill and by traversing deep valleys on the likelihood surface to avoid getting stuck on a local maximum (Kirkpatrick et al. 1983; Goffe et al. 1994). It has been proved to be highly powerful in pedigree reconstruction (Wang 2004; Wang and Santure 2009) from genotype data, which is probably more difficult than population structure reconstruction (i.e., clustering analysis) because the genetic structure (i.e., sibship) of the former is, in general, more numerous, more complicated with hierarchy, and smaller (thus more elusive and more difficult to define) than that in the latter. Herein I propose a SA algorithm for a population clustering analysis, as detailed in Supplementary Appendix 1.

### Admixture analysis

Under the mixture model, the above clustering analysis partitions the *N* sampled individuals into a predefined *K* clusters, each representing a source population. The properties (e.g., genetic diversity) of and the relationships (e.g., *F*_*ST*_) among these populations can be learnt from the inferred clusters. However, the clustering results are accurate only when the mixture model is valid. For a sample containing a substantial proportion of highly admixed individuals (i.e., who have recent ancestors from multiple source populations), the clustering results are just approximations. In such a case, the admixture model is more appropriate and can be used to refine the mixture analysis results by inferring the admixture proportions (or ancestry coefficients) of each sampled individual.

Under the admixture model (Pritchard et al. 2000), an individual *i*’s ancestry (or admixture proportions) can be characterised by a vector **q**_*i*_ = {*q*_*i*1_, *q*_*i*2_, …, *q*_*iK*_}, where *q*_*ik*_ is the proportion of its genome coming from (contributed by) source population *k*. Equivalently, *q*_*ik*_ can also be taken as the probability that an allele sampled at random from individual *i* comes from source population *k*. Obviously, we have *q*_*ik*_ ≥ 0 and $\mathop {\sum}\nolimits_{k = 1}^K {q_{ik} \equiv 1}$. The overall admixture extent of individual *i* can be measured by $M_i = 1 - \mathop {\sum}\nolimits_{k = 1}^K {q_{ik}^2}$, the probability that the two alleles at a randomly drawn locus come from different source populations. Individual *i* is purebred and admixed when *M*_*i*_ = 0 and *M*_*i*_ > 0, respectively. An F_1_ and F_2_ hybrid individual *i* is expected to have *M*_*i*_ = 0.5 and *M*_*i*_ = 0.625, respectively.

The task of an admixture analysis is to infer **q**_*i*_ for each individual *i*, denoted by **Q** = {**q**_1_, **q**_2_, …, **q**_*N*_}. The log-likelihood function is

6 $${{{\mathcal{L}}}}\left( {{{{\mathbf{Q}}}},{{{\mathbf{P}}}}\left| {{{\mathbf{X}}}} \right.} \right) = \mathop {\sum}\limits_{i = 1}^N {\mathop {\sum}\limits_{l = 1}^L {\mathop {\sum}\limits_{a = 1}^2 {{{{\mathrm{Log}}}}\left( {\mathop {\sum}\limits_{k = 1}^K {q_{ik}p_{klx_{ila}}} } \right)} } }$$

Note (6) is essentially the same as those proposed in previous studies (e.g., Tang et al. 2005; Alexander et al. 2009). It assumes independence of individuals conditional on the genetic structure defined by **Q**, and independence of alleles both within and between loci. The former can be violated when the data have genetic structure in addition to the subpopulation structure defined by **Q**, such as the presence of familial structure (Rodríguez‐Ramilo and Wang 2012) or inbreeding (Gao et al. 2007) within a subpopulation. The assumption of independence among loci is violated for markers in linkage disequilibrium. It, as well as the assumption of independence between paternal and maternal alleles within a locus, is also violated due to admixture (Tang et al. 2005) or inbreeding (Gao et al. 2007). However, (6) is a good approximation and works well in general even when these assumptions are violated, as checked by extensive simulations.

If **P** were known, it would be trivial to estimate **Q** from **X**. Unfortunately, usually, the only information we have is genotype data **X**, from which we must infer *K*, **Q** and **P** jointly. Herein I modify the EM algorithm of Tang et al. (2005) to solve (6) for maximum likelihood estimates of **Q** and **P** given *K*, as detailed in Supplementary Appendix 2.

Despite essentially the same likelihood function, my EM algorithm differs from that of Tang et al. (2005) in three aspects. First, I use the clustering results of mixture model as initial values of **Q**. Even in the worst scenario of many highly admixed individuals included in a sample, the clustering results should still be much closer to the true **Q** than a random guess, as used in previous likelihood methods (Tang et al. 2005; Alexander et al. 2009). It is possible (and indeed it has been trialled) to use the results of a faster non-model based clustering method, such as *K*-means method, in place of those of the likelihood-based clustering method with simulated annealing algorithm as described above. However, such non-model based methods are less reliable and less accurate, especially in difficult situations (below). Second, rather than updating **Q** and **P** in alternation, I update **Q** to asymptotic convergence under a given **P**. I then update **P** using the converged **Q**. This two-step iteration process is repeated until the convergence of both **Q** and **P** is reached. Third, the allele frequencies for a specific individual *i* are calculated by excluding the genotypes of the individual, which are then used in the EM procedure for iteratively updating **q**_*i*_.

### Optimal K

The above-described clustering analysis and admixture analysis are conducted by assuming a given number of source populations, *K*. Apparently, different genetic structures would be inferred from the same genotype data if different *K* values are assumed. In some cases, a reasonable *K* value is roughly known. For example, individuals might be sampled from *K* known discrete locations (say, lakes), and the purposes of a structure analysis are to confirm that populations from different locations are indeed differentiated and thus distinguishable, to identify migrants between the locations, and to find out the patterns of genetic differentiations (e.g., whether isolation by distance applies or not). In many other cases, however, we may have no idea of the most likely *K* value. For example, individuals might be sampled from the same breeding or feeding ground and we wish to know how many populations are using the same ground, and to learn the properties of these populations from the individuals sampled and assigned to them. In such a situation of hidden genetic structure, we need first to identify the most likely one or more *K* values, and then investigate the corresponding structure/admixture.

Estimating the most likely *K* value from genotype data is difficult (Pritchard et al. 2000). Although many methods have been proposed and applied (see review by Wang 2019), they are all ad hoc to some extent and may be inaccurate in difficult situations such as highly unbalanced sampling from different populations and low differentiation (Wang 2019). Herein I propose two ad hoc estimators of *K* that can be calculated from the clustering analysis presented in this study. They have a satisfactory accuracy as checked by many test datasets, simulated and empirical.

The first estimator is based on the second order rate of change of the estimated log-likelihood as a function of *K* in a clustering analysis, *D*_*LK*2_. This estimator is similar in spirit to the ∆*K* method of Evanno et al. (2005), but does not use the mean and standard deviation of log-likelihood values among replicate runs (for a given *K* value) because the standard deviation (the denominator of ∆*K*) is frequently zero thanks to the convergence of our clustering analysis by the simulated annealing algorithm.

The second estimator, denoted by *F*_*STIS*_, is based on Wright (1984)’s *F*-statistics. The best *K* should produce the strongest population structure, with high differentiation (measured by *F*_*ST*_) of each inferred cluster and low deviation from Hardy-Weinberg equilibrium (measured by *F*_*IS*_) within each inferred cluster. Details of how to calculate the two estimators are in Supplementary Appendix 3.

### Simulations

To evaluate the accuracy, robustness, and computational efficiency of the new methods implemented in PopCluster in comparison with other methods, I simulated and analysed data with different population structures and sampling intensities. The simulation procedure described below is implemented in the software package PopCluster.

#### Simulation 1, small samples

A population becomes difficult to define genetically when few individuals from it are sampled and included in an admixture analysis. However, a small sample of individuals can be common in practice when, for example, archaeological samples (usually few) are used in studying ancient population structure or in studying the relationship between ancient and current populations (e.g., Lazaridis et al. 2014). In a mixed stock analysis (Smouse et al. 1990) or a wildlife forensic analysis of source populations, there might also be few sampled individuals representing a rare population. To investigate the impact of sample sizes on an admixture analysis, I simulated 10 populations in an island model with *F*_*ST*_ = 0.05. *N*_*k*_ (=2, 3, …, 10 and 20) individuals were sampled from each of the 10 populations, or 1 individual was sampled from each of the first five populations and 2 individuals were sampled from each of the last five populations (the case *N*_*k*_ = 1.5, Table 1). Other simulation parameters are summarized in Table 1.

**Table 1:** Simulation parameters.

Simulations	*K*	*F*_*ST*_	*d* (*q*_11_)	*S*	*L*	*λ*_*FS*_	*N*_*k*_
Small samples	10	0.1	0	0	1000	0	1.5, 2–40
Many populations	3, 6, 12, 25, 50, 100	0.05	0	0	1000	0	20
High admixture	3	0.1	0.025, 0.05, 0.1, 0.2, 0.4, 0.8	0	1000	0	50
Spatial admixture model	5	0.05	0.4, 0.5, 0.6, 0.7, 0.8, 0.9, 1.0	0	10000	0	100
Low differentiation	3	0.0005,0.001, 0.002, 0.004, 0.008, 0.016, 0.032	0	0	2000000, 1000000, 500000, 250000, 125000, 62500, 31250	0	50
Unbalanced sampling	3	0.1	0	0	1000	0	4, 8, 12, 16, 20, 30, 40, 50, 60, 70, 80
Relatedness	3	0.025	0	0	1000	0, 2, 4, 8, 16	50
Inbreeding	3	0.1	0	0, 0.05, 0.1, 0.2, 0.4, 0.8	1000	0	50
Computational efficiency	2,4,8,16,32,64,128,256,512	0.1	0.1	0	10000	0	10

The columns (left to right) are simulation scenarios, number of populations (*K*), differentiation (*F*_*ST*_), admixture probability *d* (see text for details) or *q*_11_ (for spatial admixture model), selfing rate (*S*), number of loci (*L*), average full-sib family size (*λ*_*FS*_), and sample sizes (*N*_*k*_) for each simulated scenario. For the set of simulations with low differentiation, I assumed various *F*_*ST*_ values and corresponding numbers of markers, with *L* = 1000/*F*_*ST*_. For unbalanced sampling, I assume *K* = 3 populations with *N*_2_ ≡ *N*_3_ ≡ 4, 8, 12, 16, 20, 24, and *N* = *N*_1_ + *N*_2_ + *N*_3_≡ 200. The extent of unbalanced sampling is measured by the sample size ratio, *N*_1_/*N*_2_. For simulating relatedness between individuals sampled from a subpopulation, I assume a Poisson distribution of fullsib size with parameter *λ*_*FS*_ (mean sibship size). When *λ*_*FS*_ = 0, no full sibling is simulated. In all non-spatial admixture simulations, I assume an island model and two codominant alleles per locus. Results of the three scenarios of relatedness, inbreeding and high admixture are included in Supplementary Appendix 4.

#### Simulation 2, many populations

Admixture becomes increasingly difficult to infer with an increasing *K*, the number of assumed populations, because the dimensions of both **Q** and **P** increase linearly with *K*. This contrasts with the number of individuals, *N*, and the number of loci, *L*, which determines the dimensions of **Q** and **P** only, respectively. Therefore, the scale of an admixture analysis, in terms of the number of parameters to be estimated, is predominantly determined by *K* rather than *N* or *L*. I simulated data with a widely variable number of populations (*K* = [6, 100]) to see if the structure can be accurately reconstructed by using relatively highly informative markers (parameters in Table 1), especially when *K* is large which is rarely considered in previous simulation studies.

#### Simulation 3, spatial admixture model

The spatial admixture model resembles isolation by distance where population structure changes gradually as a function of geographic location. Under this model, populations are not discrete as assumed by admixture models and have no recognisable boundaries, posing challenges to an admixture analysis. To simulate the spatially gradual changes in genetic structure, I assume source populations 1, 2, …, *K* are equally spaced in that order along a line (say, a river in reality). Sampled individuals 1, 2, …, *N* are also equally spaced in that order on the same line. The admixture proportions of individual *i*, **q**_*i*_ = {*q*_*i*1_, *q*_*i*2_, …, *q*_*iK*_}, being the proportional genetic contributions to *i* from source populations *k*, are a function of the individual’s proximity to these *K* source populations. Formally, we have

7 $$q_{ik} = \frac{{q_{ik}^ \ast }}{{\mathop {\sum}\nolimits_{k = 1}^K {q_{ik}^ \ast } }}$$

where

$$q_{ik}^ \ast = \left[ {1 - \left( {\frac{{i - 1}}{{N - 1}} - \frac{{k - 1}}{{K - 1}}} \right)^2} \right]^S$$

and parameter *S* is used to regulate the admixture extent of the *N* sampled individuals. Under this spatial admixture model, an individual *i*’s admixture (**q**_*i*_) is determined by its location, or the distances from the *K* source populations. The 1st and the last sampled individuals (*i* = 1, *N*) always have the least admixture, measured by $M_i = 1 - \mathop {\sum}\nolimits_{k = 1}^K {q_{ik}^2}$. *q*_11_ (=*q*_*NK*_) is always the largest among the *q*_*ik*_ values for *i* = 1, 2, …, *N* and *k* = 1, 2, …, *K*. Given a desired value of *q*_11_ and *K*, the scaler parameter *S* can be solved from the above equations. Given *K*, *N* and *S*, **q**_*i*_ of an individual *i* can then be calculated from the above equations. In this study, I simulated and analysed samples generated with parameters *K* = 5, *N* = 500, *L* = 10000 SNPs, and *q*_11_ varying between 0.5 and 1.0 (Table 1).

#### Simulation 4, low differentiation

Population structure analysis becomes increasingly difficult with a decreasing differentiation, usually measured by *F*_*ST*_, among subpopulations. Fortunately, with genomic data of many SNPs, it is still possible to detect weak and subtle population structures (Patterson et al. 2006) as demonstrated in human fine-structure analysis (e.g., Leslie et al. 2015). I simulated data with varying weak population structures (low *F*_*ST*_, Table 1) and otherwise ideal populational (only 3 equally differentiated subpopulations) and sampling conditions (i.e., a large sample of individuals per subpopulation, and many SNPs). The number of SNPs used in analyse was *L* = 1000/*F*_*ST*_ such that in principle the population structures should be inferred with roughly equal power and accuracy. Because *L* is large for low *F*_*ST*_, STRUCTURE analysis was abandoned due to computational difficulties.

#### Simulation 5, unbalanced sampling

Samples of individuals from different source populations are rarely identical in size in practice. Frequently, different source populations are represented by different numbers of individuals in a sample. The impact of unbalanced sampling and how to mitigate it in applying STRUCTURE have been investigated (e.g., Puechmaille 2016; Wang 2017). Similar problems exist for other admixture or clustering analysis methods but have not been studied yet. The same population structure and unbalanced sampling schemes (see parameters in Table 1) used in Wang (2017) were used to simulate data, which were then analysed by various methods to understand their robustness to unbalanced sampling.

#### Simulation 6, computational efficiency

Samples from a variable number of populations (Table 1) were analysed by the four programs on a linux cluster to compare their computational efficiencies. Each program uses a single core (no parallelisation) of a processor (Intel Xeon Gold 6248 2.5 GHz) for a maximal allowed time of 48 or 72 (when *K* = 1024 only) hours. Default parameter settings are used for all four programs. For STRUCTURE, both burn-in and run lengths were set to 10^4^, although much higher burn-in is required for convergence when *K* is large (say *K* > 20). The running time for STRUCTURE is thus conservative, especially when *K* is not small.

Further simulations were conducted to investigate the effects of high admixture and the presence of familial relationships and inbreeding on the relative performance of different admixture analysis methods, as detailed in Supplementary Appendix 4.

In all simulations except for the spatial admixture model, I assumed a population with *K* discrete subpopulations in Wright’s (1931) island model in equilibrium among mutation, drift and migration. For a locus *l* (=1, 2, …, *L*) with *J*_*l*_ alleles, allele frequencies of the ancestral population, **p**_0*l*_ = {*p*_0*l*1_, *p*_0*l*2_, …, $p_{0lJ_l}$}, were drawn from a uniform Dirichlet distribution, ${{{\mathcal{D}}}}\left( {\lambda _1,\lambda _2, \ldots ,\lambda _{J_l}} \right)$ where *λ*_*j*_ = 1 for *j* = 1, 2, …, *J*_*l*_. Given **p**_0*l*_, allele frequencies of subpopulation *k* (=1, 2, …, *K*), **p**_*kl*_ = {*p*_*kl*1_, *p*_*kl*2_, …, $p_{klJ_l}$}, were drawn from a uniform Dirichlet distribution, ${{{\mathcal{D}}}}\left( {\lambda _1,\lambda _2, \ldots ,\lambda _{J_l}} \right)$, where $\lambda _j = ( {\frac{1}{{F_{ST}}} - 1} )p_{0lj}$ for *j* = 1, 2, …, *J*_*l*_ (Nicholson et al. 2002; Falush et al. 2003). Given **p**_*kl*_ and the admixture proportion **q**_*i*_ of individual *i*, two alleles at locus *l* were drawn independently to form the individual’s genotype. The multilocus genotype of an individual was obtained by combining single locus genotypes sampled independently, assuming linkage equilibrium. *N*_*k*_ individuals were drawn at random from population *k* (= 1, 2, …, *K*), which were then pooled and subjected to a structure analysis.

For the spatial population and sampling model, allele frequencies at a locus *l*, **p**_0*l*_ and **p**_*kl*_, are generated as before, assuming *F*_*ST*_ = 0.05 among *K* = 5 subpopulations. A number of *N* = 500 individuals, equally spaced on the line between source populations 1 and 5, are sampled. The admixture proportion of individual *i*, **q**_*i*_, is determined by its location, calculated by Eq. (7). Given **p**_*kl*_ and **q**_*i*_, the multilocus genotype of individual *i* is simulated as described above.

For each parameter combination, 100 replicate datasets were simulated, analysed and assessed for estimation accuracy. Each dataset was analysed for admixture by different methods (see below for details) with an assumed *K* as used in simulations. I did not consider estimating the optimal *K* by analysing a simulated dataset in a range of possible *K* values. This is because, like previous studies (e.g., Pritchard et al. 2000; Alexander et al. 2009), I am more concerned with admixture inference under a given *K*, which is important of itself and forms the basis for inferring the optimal *K* as well. This is also because it is almost impossible computationally to estimate the optimal *K* for so many replicate datasets and so many parameter combinations in a large-scale simulation study like the present one, even when using large computer clusters. The optimal *K* was estimated for several empirical datasets (below).

### Measurement of accuracy

Inference accuracy could be assessed by comparing, for each individual *i*, the agreement between simulated ancestry coefficients, **q**_*i*_, and estimated ancestry coefficients, $\widehat {{{\mathbf{q}}}}_i$, obtained by an admixture analysis assuming the true/simulated subpopulation number *K*. Because the reconstructed populations are labelled arbitrarily (Pritchard et al. 2000), no meaningful results can be gained by comparing **q**_*i*_ and $\widehat {{{\mathbf{q}}}}_i$ directly, however. It is possible to relabel the reconstructed populations and find the labelling scheme that has the maximum agreement between **q**_*i*_ and $\widehat {{{\mathbf{q}}}}_i$ as the measurement of accuracy. However, there are *K*! possible labelling schemes, making the approach difficult to calculate when *K* is large (say, *K* > 50).

The labelling becomes irrelevant when pairs of individuals are considered for the co-assignment probabilities (or coancestry) (Dawson and Belkhir 2001). I calculate and use the average difference between simulated and estimated coancestry for pairs of sampled individuals to measure the average assignment error, AAE (Wang 2017),

8 $$AAE = \left( {\frac{1}{{N\left( {N - 1} \right)/2}}\mathop {\sum}\limits_{i = 1}^N {\mathop {\sum}\limits_{j = 1 + 1}^N {\left( {\mathop {\sum}\limits_{k = 1}^K {q_{ik}q_{jk}} - \mathop {\sum}\limits_{k = 1}^K {\widehat q_{ik}\widehat q_{jk}} } \right)^2} } } \right)^{1/2}.$$

The minimum value of AAE is 0, when ancestry (admixture) is inferred perfectly. The maximum value is 1, when there are no admixed individuals in the sample, individuals from the same source population are always assigned to different populations and individuals from different source populations are always assigned to the same population. It is worth noting that the minimum AAE value of 0 is always possible for any population structure. However, the maximum value varies and can be much smaller than 1, depending on the actual underlying population structure. With an increasing *K* value or increasing admixture (i.e., *q*_*ik*_→1/*K* for any individual *i*), the maximum value of AAE tends to decrease. For this reason, AAE cannot be compared fairly between different genetic structures (e.g., different *K* values, different actual **Q** for a given *K*, or different sizes of subsamples from the source populations) for measuring the relative inference qualities. However, it can always be used to compare the accuracy of different inference methods for a given simulated genetic structure and a given sample.

### Analysis of real datasets

#### An ant dataset

It was originally used in a study of the mating system of an ant species, *Leptothorax acervorum* (Hammond et al. 2001). Ten sampled colonies, A, B, C, D, E, F, G, H, I, and J, contribute respectively 9, 7, 47, 45, 45, 45, 45, 45, 44, and 45 diploid workers to a sample of 377 individuals. For this species, we know that each colony is headed by a single diploid queen mated with a single haploid male. Therefore, workers from the same colony are full-sibs and workers from different colonies are non-sibs. Each sampled worker was genotyped at up to 6 microsatellite loci, which have 3 to 22 alleles per locus observed in the 377 individuals. This dataset was analysed to reconstruct the genetic structure of the sample, which actually is the family structure. ADMIXTURE and sNMF cannot handle multiallelic marker data and therefore only STRUCTURE and PopCluster are used for analysing this dataset.

For STRUCTURE, I used the default parameter settings, except for the burning-in and run lengths which were both set to 10^5^ to reduce the risk of non-convergence. Two analyses were conducted. First, optimal *K* values were determined using three estimators (Wang 2019) calculated from STRUCTURE outputs, and using the *D*_*LK*2_ estimator of PopCluster. For this *K* estimation purpose, 20 replicate runs for each possible *K* value in the range [1, 15] were conducted by both STRUCTURE and PopCluster. Second, assuming *K* = 10, a number of 100 replicate runs (each with a distinctive seed for the random number generator) were conducted by both STRUCTURE and PopCluster to investigate their convergence.

#### An Arctic charr dataset

Shikano et al. (2015) sampled 328 Arctic charr individuals from 6 locations in northern Fennoscandia: two lakes (Galggojavri and Gallajavri) and one pond (Leenanlampi) in the Skibotn watercourse drain into the Atlantic Ocean and three lakes (Somasjärvi, Urtas-Riimmajärvi and Kilpisjärvi) in the Tornio-Muoniojoki watercourse drain into the Baltic Sea. Individuals were genotyped at 15 microsatellite loci to study the genetic structure and demography. The data were again analysed by STRUCTURE and PopCluster but not by ADMIXTURE and sNMF because the markers are multiallelic. I conducted two separate analyses of the genotype data. First, I estimated the most likely *K* value by each program, making 20 replicate runs with each *K* value in the range [1, 10]. Second, I investigated the convergence of each program by conducting 100 replicate runs of the data at *K* = 6. STRUCTURE analyses were run with default parameter settings except for both burn-in and run lengths being 10^5^.

#### A human SNP dataset

Using FRAPPE (Tang et al. 2005), Li et al. (2008) studied the world-wide human population structure represented by 938 individuals sampled from 51 populations of the Human Genome Diversity Panel (HGDP). Each individual was genotyped at 650000 common SNP loci. The data were expanded to include genotypes of 1043 individuals at 644258 SNPs, available from http://www.cephb.fr/en/hgdp_panel.php#basedonnees. In this study, the expanded data were comparatively analysed by PopCluster, ADMIXTURE, and sNMF, assuming *K* = 7 clusters (regions) as in the original study (Li et al. 2008). STRUCTURE was too slow to analyse this big dataset and thus it was abandoned.

#### The human 1000 genomes phase I dataset

The dataset (Abecasis et al. 2012), available from https://www.internationalgenome.org/data/, has 1092 human individuals sampled from 14 populations across all continents, with each individual having 38 million SNP genotypes. After removing monomorphic loci (note, no pruning was applied regarding missing data, minor allele frequency and linkage disequilibrium, in contrast to other studies), genotypes at a number of *L* = 38035992 SNPs were analysed by PopCluster and sNMF, assuming *K* = 9 clusters (regions). Both STRUCTURE and ADMIXTURE were too slow to analyse this huge dataset and thus were abandoned. No attempts are made to find the optimal *K* for this dataset as done for the ant and Arctic charr datasets, because too much computational time is required for PopCluster or sNMF to analyse the data with a number of replicate runs at each of a number of *K* values even when using a large cluster, and there might be multiple *K* values that explain the data equally well (at different spatial and time scales). For a better understanding of the world-wide human population genetic structure, the data should be analysed at least with one replicate under each of a number of possible *K* values, say *K* = [5, 12], to reveal and compare the genetic structure. This study analysed the data at a single *K* = 9 for the purpose of demonstrating the capacity of different methods, and comparing the admixture estimates of PopCluster and sNMF at this particular value of *K*. Because of the incompleteness of the analysis, the biological interpretations of the results should be taken with caution.

### Comparative analyses by different software

I compared the accuracy and computational time of STRUCTURE (Pritchard et al. 2000; Falush et al. 2003), ADMIXTURE (Alexander et al. 2009), sNMF (Frichot et al. 2014) and PopCluster in analysing both simulated and empirical datasets described above. Quite a few other model-based methods implemented in various software exist. I choose STRUCTURE and ADMIXTURE because they are the most popular model-based admixture analysis methods used for small and large datasets, respectively. I also choose sNMF because it is a very fast model-based method that works for huge datasets for which other methods, such as ADMIXTURE, fail to run or take unrealistically too much time to run.

STRUCTURE can handle both diallelic (such as SNPs) and multiallelic (such as microsatellites) markers, but runs too slowly to analyse large datasets with many markers, many individuals, or many populations. It was therefore used to analyse all simulated and empirical datasets with no more than 10000 loci. The default parameter setting was used for most datasets, with a burn-in length of 10^4^ and a run length of 10^4^ iterations. For better convergence, the burn-in and run lengths were increased to 10^5^ iterations for analyses involving a large number of simulated populations (say, when *K* ≥ 10) or for analyses of empirical datasets. For unbalanced sampling, the alternative ancestry model instead of the default model was used by setting POPALPHAS = 1.

Both ADMIXTURE and sNMF were developed specifically for diallelic markers and could not analyse multiallelic marker data. In this study, they were used to analyse SNP data only. For the human 1000 genome phase I data, however, ADMIXTURE could not complete the analysis within a realistic period of time (72 h, the maximum allowed in the linux cluster used for the analysis) even when the maximal number of parallel threads were used. Therefore, only sNMF and PopCluster were used to analyse this dataset.

To understand the relative computational efficiency and how much speedup can be gained by parallelisation, ADMIXTURE, sNMF and PopCluster were used to analyse the HGDP dataset and the 1000 genome dataset, by using a variable number of parallel threads on a linux cluster with many nodes, each having 32 cores. The maximum wall clock time allowed for a job on the cluster is 48 h.

## Results

### Simulation 1, small samples

**Fig. 1:**
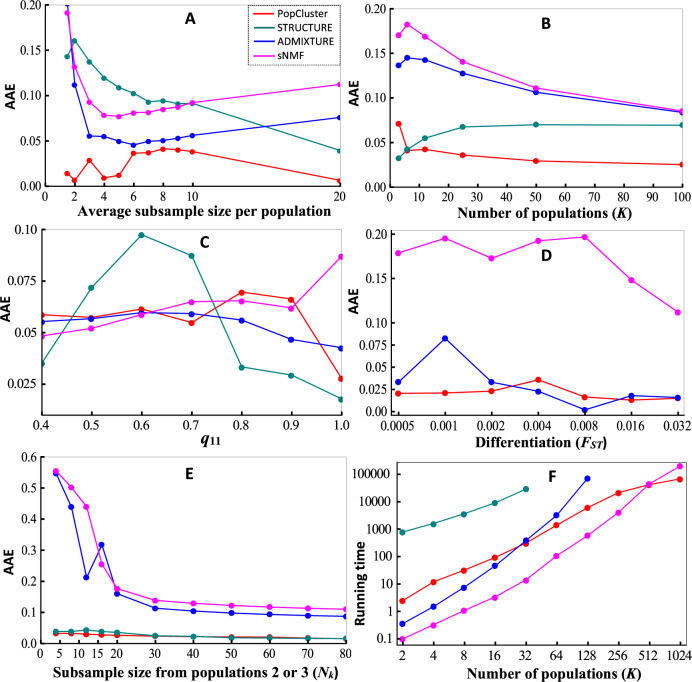
Simulation results. **A** Average assignment error, AAE, as a function of the average subsample size (1.5, 2, 3, …, 10 and 20 individuals) from each of *K* = 10 populations. The populations were assumed to have *F*_*ST*_ = 0.1 in the island model, and each sampled individual was genotyped at 1000 SNP loci. **B** AAE as a function of the number of populations (*K*). 20 individuals were sampled from each of *K* populations simulated with *F*_*ST*_ = 0.05 in the island model, and each sampled individual was genotyped at 1000 SNP loci. **C** AAE as a function of *q*_11_ in spatial admixture model. 500 individuals were sampled from *K* = 5 populations simulated with *F*_*ST*_ = 0.05 in spatial admixture model, and each sampled individual was genotyped at 10000 SNP loci. **D** AAE as a function of genetic differentiation between populations (*F*_*ST*_). *K* = 3 populations with varying *F*_*ST*_ (on *x* axis) in the island model were simulated, 50 individuals were sampled from each population, and each sampled individual was genotyped at a number of *L* = 1000/*F*_*ST*_ SNP loci. **E** AAE as a function of the subsample size of population 2 or 3 (*N*_*k*_). The island model of *K* = 3 populations with *F*_*ST*_ = 0.1 was simulated. A subsample of *N*_*k*_ (*x* axis) individuals was sampled from population 2 or 3, and a subsample of 300−2*N*_*k*_ individuals was sampled from population 1. Each individual was genotyped at a number of *L* = 1000 SNP loci. **F** Running time (seconds) as a function of the number of populations (*K*). The island model of *K* = [2, 1024] populations with *F*_*ST*_ = 0.1 was simulated. 10 individuals were sampled from each population and genotyped at *L* = 10000 SNP loci.

STRUCTURE performs poorly when a sample contains a small number of individuals drawn from each population (Fig. 1A). When each population is represented by just a few individuals (say, 10 or fewer), STRUCTURE is less accurate in inferring individual admixture proportions, making more errors than other methods. However, with an increasing sample size for each population, it becomes more accurate than ADMIXTURE and sNMF. Across the entire range of sample sizes considered in the simulation, ADMIXTURE is more accurate than sNMF, and PopCluster is the most accurate among the four methods.

The poor performance of STRUCTURE, ADMIXTURE, and sNMF when each population is represented by just a few individuals is caused by the difficulty in delineating a population by its allele frequencies. In such a situation, the three methods tend to make sporadic population splits and merges, to infer extensive admixture or both, as shown in Supplementary Appendix 5 for a particular simulated dataset.

### Simulation 2, many populations

In the range of simulated number of populations (*K* from 3 to 100), STRUCTURE is the most accurate when *K* is small (*K* < 6), but quickly PopCluster becomes the most accurate when *K* becomes medium or large (Fig. 1B). ADMIXTURE and sNMF are consistently less accurate than the other two methods in the entire range of *K* = [3100].

ADMIXTURE and sNMF are less accurate than STRUCTURE and PopCluster because they tend to infer too much admixture, as shown in Supplementary Appendix 6 for a particular simulated dataset with *K* = 10.

### Simulation 3, spatial admixture model

The assignment errors of the four methods for different *q*_11_ values used in simulations under the spatial admixture model are shown in Fig. 1C. STRUCTURE is the most accurate method when the populations are highly admixed (*q*_11_ < 0.45) or lowly admixed (*q*_11_ > 0.75), but is the least accurate when the populations are mediumly admixed (0.45 < *q*_11_< 0.75). PopCluster, ADMIXTURE and sNMF have similar performance when *q*_11_ is not very high. However, as *q*_11_ increases to 1, PopCluster and sNMF become the most and least accurate one of the three methods, respectively. The simulated and estimated admixture of a particular dataset generated with *q*_11_ = 0.9 is shown in a bar chart in Supplementary Appendix 7.

### Simulation 4, low differentiation

At low differentiation and in otherwise ideal populational and sampling situations, both PopCluster and ADMIXTURE can infer structure accurately (Fig. 1D). In contrast, sNMF is inaccurate, producing much higher inference errors than other methods. sNMF overestimates admixture, as shown by the admixture bar chart for a particular dataset in Supplementary Appendix 9. Both ADMIXTURE and sNMF also have a convergence problem, as exemplified by Figs. A9–1.

### Simulation 5, unbalanced sampling

Using the scaling scheme (Eq. 5) in PopCluster and the alternative ancestry prior in STRUCTURE, both methods yield accurate structure inferences when population representations in the sample are unbalanced (Fig. 1E). In contrast, inferences from ADMIXTURE and sNMF are very inaccurate when sampling is highly unbalanced. As a confirmation of the importance of appropriate scaling and prior, PopCluster without the scaling scheme and STRUCTURE with the default ancestry prior yield similar results to those of ADMIXTURE and sNMF (not shown in Fig. 1E for clarity). The inaccuracy of ADMIXTURE and sNMF is caused by their overestimation of admixture, as shown in Supplementary Appendix 8 for a particular dataset.

### Other simulations

Overall, PopCluster and STRUCTURE have a similar performance and are more accurate than sNMF and ADMIXTURE when selfing is present and when admixture occurs at various extents (Supplementary Appendix 4). However, STRUCTURE is sensitive to the presence of sibship structure in a sample, and becomes the least accurate method when large full sib families are included in a sample.

### Simulation 6, computational efficiency

STRUCTURE is slower than the other programs by roughly 1000 times (Fig. 1F), despite of the use of a short burn-in and run length (=10000) which could be insufficient for convergence when *K* is not small. ADMIXTURE runs faster than PopCluster only when *K* < 32. Its running time per iteration increases quadratically with *K* (Alexander et al. 2009) and therefore it becomes slower than PopCluster when *K* > 32. Within the range of *K* = [2512], sNMF is the fastest among the four programs. However, its computational efficiency advantage over PopCluster diminishes with an increasing *K*. It is overtaken by PopCluster when *K* > 512. Within the 48 h limits, the maximal number of populations that can be analysed successfully is 32 for STRUCTURE, 128 for ADMIXTURE, and 512 for both PopCluster and sNMF. For *K* = 1024, the analyses by PopCluster and sNMF were conducted on a linux cluster with a maximal job duration of 72 h. The computational efficiency of PopCluster over other programs becomes more prominent with an increasing number of markers. At *K* = 512, PopCluster and sNMF have a similar running time (Fig. 1F). However, when the number of loci is increased to 1 million, PopCluster and sNMF take 16 and 26 h respectively to complete the analysis using 36 cores in parallel.

### Analysis of the ant dataset

**Fig. 2:**
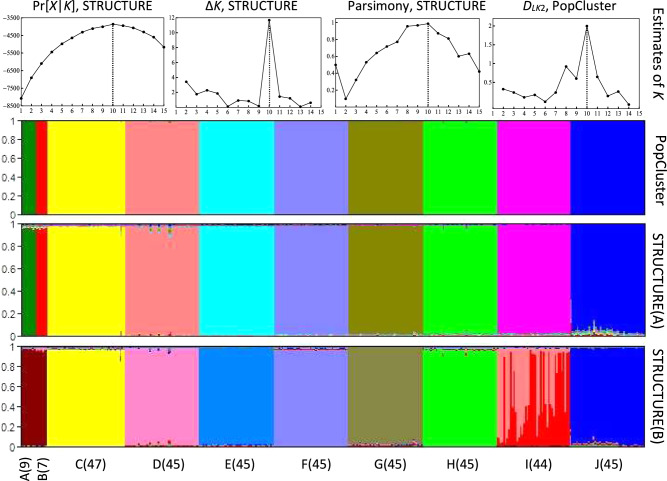
Analysis of the population structure of 377 ant workers sampled from 10 colonies and genotyped at 6 microsatellites. The first row shows the optimal *K* (indicated by vertical dotted lines) estimated by three different estimators using STRUCTURE outputs, and by the *D*_*LK*2_ estimator of PopCluster. The other rows show individual ancestry inferences by PopCluster (the 2nd row) and STRUCTURE (the 3rd and 4th rows) assuming the optimal *K* = 10. Each individual is represented by a thin vertical line partitioned into *K* = 10 coloured segments that represent the individual’s estimated membership fractions in *K* clusters. The 10 colonies (sampled numbers of workers) are shown on the *x* axis. The 3rd (A) and 4th (B) rows show the results from two replicate STRUCTURE runs yielding a higher and lower estimated probability of data.

Using genotype data of only six microsatellites, both PopCluster and STRUCTURE recovered the colony structure of the ant sample. First, different *K* estimators using STRUCTURE outputs and the *D*_*LK*2_ estimator of PopCluster yield the same result, *K* = 10, which agrees with the known number of colonies represented by the 377 sampled workers. Second, both PopCluster and STRUCTURE assigned these 377 individuals into 10 populations corresponding to the 10 sampled colonies (Fig. 2). Each individual was inferred to have no or little admixture, with its ancestry coming almost exclusively from a single source population (colony). Relatively, STRUCTURE yields slightly more admixture than PopCluster. Analyses by PopCluster conducted with both scaling and no scaling yielded the same results.

STRUCTURE has a convergence problem for this dataset. Among the 100 replicate runs with *K* = 10, 91 runs correctly recovered the colony structure with minor differences in admixture proportion estimates, and in estimated log probability of data, LnPrb (maximum = −3758.1, minimum = −3768.4). Nine runs did not reconstruct the colony structure correctly. They either merged the two small colonies (with 7 and 9 workers) and thus produced 9 clusters, or one of the large colonies showed extensive admixtures, or both (see one example in Fig. 2). The 9 runs had much smaller LnPrb values, from −3822.2 to −4163.7. For the example shown in the lower panel of Fig. 2, the LnPrb value is −3843.4. In contrast, PopCluster converges reliably for this dataset, with all 100 replicate runs yielding the same colony structure with the same maximum likelihood value.

### Analysis of the Arctic charr dataset

**Fig. 3:**
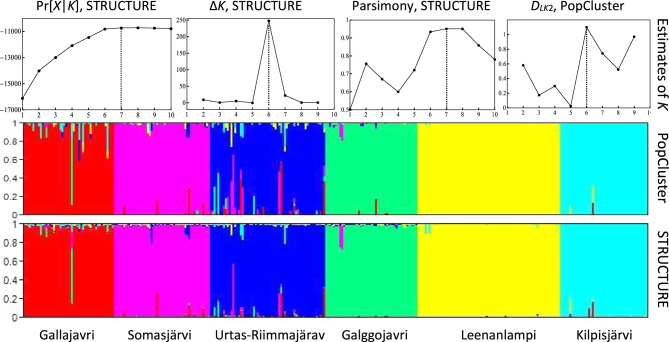
Analysis of the population structure of a sample of 328 Arctic charr individuals genotyped at 15 microsatellites. The upper panel shows the optimal *K* (indicated by vertical dotted lines) estimated by three different estimators using STRUCTURE outputs, and by the *D*_*LK*2_ estimator of PopCluster. The middle and lower panels show individual ancestry inferences by PopCluster and STRUCTURE assuming *K* = 6. Each individual is represented by a thin vertical line partitioned into *K* = 6 coloured segments that represent the individual’s estimated membership fractions in the *K* clusters. Individuals were ordered according to the sampling locations/populations shown on the *x* axis.

Similar results are obtained from PopCluster and STRUCTURE (Fig. 3). At *K* = 6, both programs reconstructed 6 clusters, each consisting mostly of individuals from a single sampling location only. The results shown in Fig. 3 are also very similar to those in the original study (Shikano et al. 2015) using both STRUCTURE (but the correlated allele frequency model) and BAPS (Corander et al. 2003). Different from the ant data, STRUCTURE converges reliably for this charr dataset at *K* = 6, with all 100 replicate runs yielding essentially the same results, with minor differences in LnPrb (from −10790.9 to −10820.9) and in individual admixture proportion estimates. PopCluster again produced the same results (maximum likelihood and admixture) among the 100 replicate runs.

Also different from the ant data, reliable estimates of the optimal number of populations, *K*, are difficult to obtain from this Arctic charr dataset. The Δ*K* estimator for STRUCTURE and the *D*_*LK*2_ estimator of PopCluster yield an estimate of *K* = 6, consistent with the number of sampling locations. The other two estimators for STRUCTURE give *K* = 7, although its supporting evidence is not much stronger than that at either *K* = 6 or *K* = 8 (Fig. 3).

### Analysis of the human SNP dataset

**Fig. 4:**
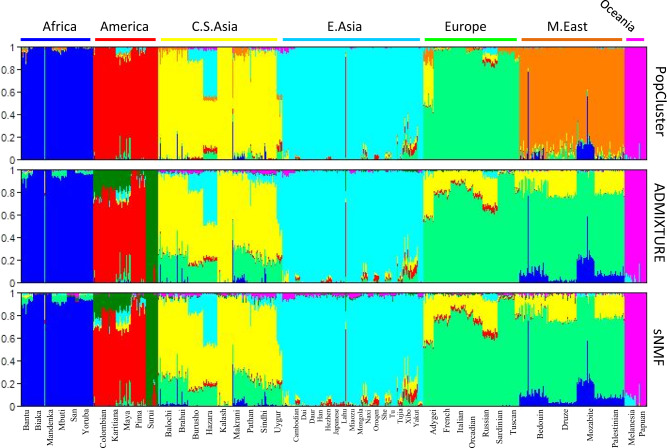
PopCluster (upper), ADMIXTURE (middle) and sNMF (lower) inferred population structures of a world-wide sample of 1043 human individuals genotyped at 644258 SNPs (644199 polymorphic). Each individual is represented by a thin vertical line partitioned into *K* = 7 coloured segments that represent the individual’s estimated membership fractions in the *K* clusters. Individuals were ordered according to the sampling locations/populations shown on the *x* axis.

ADMIXTURE and sNMF yield almost identical results (Fig. 4). They merged Middle East and Europe into a single cluster. The main difference between the two populations is that Middle East has a small fraction of African ancestry, while Europe has none. Surui people were split from the Americans to form a separate one of the *K* = 7 clusters. These results contrast with those from PopCluster, which partitioned the 1043 people into 7 clusters that correspond nicely to the sampling regions of Africa, America, Central South Asia, East Asia, Europe, Middle East, and Oceania. PopCluster results are in broad agreement with the original analysis results of Li et al. (2008) using FRAPPE.

### Analysis of the human 1000 genome phase I dataset

**Fig. 5:**
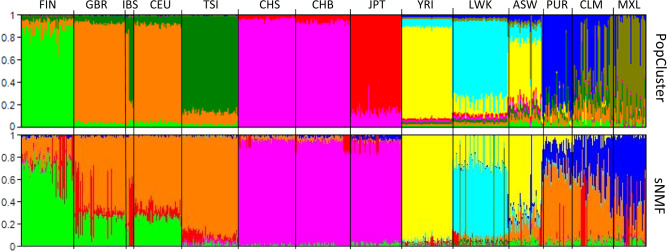
PopCluster (upper) and sNMF (lower) inferred population structures of a world-wide sample of 1092 human individuals genotyped at 38035992 SNPs (human 1000 genomes project, Phase I data). Each individual is represented by a thin vertical line partitioned into *K* = 9 coloured segments that represent the individual’s estimated membership fractions in the *K* clusters. Individuals were ordered according to the sampling locations/populations shown on the *x* axis (top). The 14 sampling populations (sample sizes) are: FIN, Finish (93); GBR, British (89); IBS, Spanish (13); CEU, CEPH Utah residents (85); TSI, Tuscan (98); CHS, Southern Han Chinese (100); CHB, Han Chinese (97); JPT, Japanese (89); YRI, Yoruba (99); LWK, Luhya (97); ASW, African-American (61); PUR, Puerto Rican (55); CLM, Colombian (60); MXL, Mexican–American (66).

At *K* = 9, sNMF infers much more admixture than PopCluster (Fig. 5). European populations, except for the Italians (TSI), are all highly admixed, according to sNMF. However, these populations are inferred to have much less admixture by PopCluster. The Japanese and the Chinese form a single cluster by sNMF, but are separated into two clearly different clusters by PopCluster. The Japanese share more ancestry with northern Chinese than southern Chinese according to PopCluster, but this trend is invisible from sNMF.

### Efficiency of parallel computation

Using the same number of cores, sNMF is the fastest and ADMIXTURE is the slowest for analysing the human HGDP dataset (Table 2). All three methods take less time to complete the analysis with the use of an increasing number of parallel threads. PopCluster benefits more from the parallelisation than sNMF and ADMIXTURE. All methods run slower with an increasing *K*. However, PopCluster is much less affected by an increasing *K* than the other methods, as expected because only a small fraction of variables need to be updated in the clustering iterations of PopCluster.

**Table 2:** Runtime for analysing two human datasets.

		Number of parallel threads										
Dataset (*K*)	Methods	1	2	4	8	16	32	64	128	256	512	1024
HGDP (7)	sNMF	28	6	12	6	5						
	Admixture	256	189	147	183	165						
	PopCluster	352	89	54	25	14						
HGDP (12)	sNMF	36	30	15	10	13						
	Admixture	601	326	235	193	185						
	PopCluster	355	173	81	43	19						
HGDP (24)	sNMF	48	68	28	28	15						
	Admixture	1573	987	608	446	325						
	PopCluster	394	212	90	43	19						
1000Genome (9)	sNMF	974	789	497	258	197	118	-	-	-	-	-
	PopCluster	*	*	*	2316	1897	1328	929	714	420	276	193

Runtime is in minutes of wall clock time. The human HGDP dataset (1043 individuals, 644258 SNPs) was analysed by assuming *K* = 7, 12, and 24 with [1,16] parallel threads. The human 1000 genome phase I dataset was analysed assuming *K* = 9 by sNMF with [1,32] parallel threads, and by PopCluster with [8,1024] parallel threads. “-“ and “*” mean no run was conducted because of the constraint in the number of threads in a node (sNMF) and in wall clock time allowed for a job (PopCluster), respectively.

When *K* = 9 is assumed for analysing the 1000 genome dataset by the same number of parallel threads, ADMIXTURE fails to finish the run within the maximally allowed 48 h on the cluster, and sNMF runs much faster than PopCluster (Table 2). However, sNMF must use parallel threads with shared memory. As a result, the maximum number of parallel threads that can be used by sNMF is only 32, the number of cores of a node in the cluster used for analysing the data. PopCluster uses both MPI and openMP to exploit parallelisation with both shared and distributed memory. It can therefore use all of the cores and all of the distributed memories across the nodes in a computer cluster for parallel computation and storage of data. PopCluster using 1024 threads runs as fast as sNMF using 16 threads for this dataset with *K* = 9. However, at higher *K* values (say, *K* = 50), PopCluster runs faster than sNMF using the same number of threads (data not shown). It can also handle huge datasets (say, terabytes of genotype data) that are impossible to fit into any shared memory but can be subdivided and fitted into distributed memories in a cluster.

## Discussions

In this study, I proposed a new method to make unsupervised population structure inference from a sample of multilocus genotypes only. As verified by analysing simulated and empirical datasets, it is advantageous over the most popular Bayesian and likelihood admixture analysis methods. It is the first model-based admixture analysis method that can handle both small multiallelic marker datasets (e.g., a few microsatellites) and huge diallelic marker datasets (e.g., millions of SNPs). STRUCTURE, having elaborated models of, among others, prior allele frequency distributions and prior ancestry distributions, is accurate, especially in difficult situations such as low differentiation and unbalanced sampling. However, it is computationally too demanding to analyse genomic data. Even a dataset with a few thousands of SNPs poses a serious challenge for STRUCTURE to analyse, especially in determining *K* as quite a few replicate runs for each of a number of possible *K* values need to be conducted (e.g., Evanno et al. 2005). Methods capable of handling genomic data, such as ADMIXTURE (Alexander et al. 2009), sNMF (Frichot et al. 2014) and TeraStructure (Gopalan et al. 2016), use much simpler methods to allow for the adoption of faster algorithms. As a result, they are fast and can handle large datasets, but apply to diallelic markers (SNPs) only and have compromised accuracy in difficult situations such as low differentiation or unbalanced sampling (Fig. 1). Furthermore, ADMIXTURE cannot handle extremely large datasets such as the human 1000 genomes phase I genotype data (with 38 million SNPs of 1092 individuals), and it becomes rather slow when many populations are assumed (Fig. 1F). sNMF has the capacity to run such large datasets, and is the fastest when *K* is not big (say, *K* < 100) and *L* (number of loci) is not extremely large (say, <10000000). Otherwise, it runs slower than PopCluster (Fig. 1F). I have also simulated a large dataset of 10^8^ individuals sampled from 100 populations, with each individual genotyped at 100 loci. While both ADMIXTURE and sNMF fail to run (with a fragmentation error) this huge sample at *K* = 100 on a linux cluster with 192GB RAM and 36 cores per node, PopCluster can successfully analyse the data, although taking a long time (4 weeks) on a laptop (running Windows 10) with an eight-core cpu and 64GB RAM. I also generated a simulated dataset with 4000 individuals, each genotyped at 50000000 SNP loci. Both ADMIXTURE and sNMF fail to run (with a segmentation fault) the dataset on the same linux cluster, even when a small number of *K* = 2 populations is assumed. PopCluster completed analysing the dataset using 36 cores of a node of this cluster in 2 days.

The computational efficiency of PopCluster benefits mainly from its two-step procedure. The first step makes a clustering analysis by assuming the mixture model (i.e., no admixture or hybridisation). It is fast, because each iteration for clustering reconfiguration involves the use and update of only a small fraction of the variables. Most often a proposal changes the cluster membership of one individual only, such that allele frequencies of only two clusters need to be recalculated. The larger is the number of clusters assumed in an analysis, the more efficient is this clustering method. The second step of admixture analysis is also fast, because the clustering analysis results (cluster membership and allele frequencies) are adopted as the initial configuration which is already close to the optimum. Comparative analyses of simulated and empirical data show that PopCluster is about two orders of magnitude faster than STRUCTURE. It runs slower than ADMIXTURE and sNMF when datasets are small or medium (say, less than 1 million SNPs, individuals in hundreds or thousands), or when the number of assumed populations are not large. For a large dataset such as the human 1000 genome phase I dataset, ADMIXTURE fails to run. While sNMF still runs faster than PopCluster when *K* is assumed to be around 7, it runs slower than PopCluster when larger *K* value (say, *K* = 30) is assumed. For even larger (say, close to terabyte) datasets, sNMF no longer runs and aborts with a segmentation fault. PopCluster uses MPI for parallel computation. It is not constrained by data size because data can be partitioned and loaded into distributed memories of a cluster with many nodes. With access to a decent computer cluster, PopCluster can handle terabytes (say, 1 million SNPs for 1 million individuals) of genotype data. Although quite a few methods, such as ADMIXTURE (Alexander et al. 2009), sNMF (Frichot et al. 2014) and TeraStructure (Gopalan et al. 2016), can analyse population structure in parallel to speed up the process, they all use parallel threads with shared memory, limiting the number of cores and the memories that can be used in a computer cluster. PopCluster is probably the first method that can use the full CPU and memory resources available to a cluster for population structure analysis.

Extensive simulation and empirical data analyses show that overall PopCluster is more accurate than ADMIXTURE and sNMF, and compares favourably over STRUCTURE, especially when few individuals are sampled from a population (Fig. 1A), many populations are sampled (Fig. 1B), sampling is highly unbalanced across populations (Fig. 1E), and inbreeding or family structures are present in a sample (Supplementary Appendix 4). PopCluster converges more reliably than STRUCTURE, as demonstrated by the ant data. While 91 of 100 replicate STRUCTURE analyses of the data at *K* = 10 yield similarly high likelihood values and similar admixture inferences, the remaining 9 replicates yield lower likelihood values and more admixture. In contrast, all 100 replicate PopCluster analyses of the data yield the same maximum likelihood and the same admixture estimates. With an increasing amount of data and increasing complexity (determined by the number and differentiation patterns of source populations, presence of hierarchical or family structures) of population structure, the risk of non-convergence increases. The better performance (accuracy and convergence) of PopCluster comes mainly from the simulated annealing (SA) algorithm adopted in clustering analysis. It is well known that SA is a global optimisation algorithm suitable for solving very large and complex systems (Kirkpatrick et al. 1983), such as our population structure analysis which involves a huge number (in millions) of variables. My simulation and empirical data analyses show SA frequently converges very well, especially when the population structure is strong, and marker information is ample. In difficult situations such as insufficient marker information or an assumed *K* value different from the truth, different replicate runs of PopCluster may yield different clustering configurations and admixture inferences with slightly different likelihood values. However, these configurations are usually very close to each other, with just a few differences in individual memberships.

Estimating *K* is much more difficult than estimating individual ancestry or admixture under a given *K*, when the information available to an analysis is genotype data only. There may exist several different *K* values that could explain the data and describe population structure closely or equally well. For example, a population might split into subpopulations A and B in the past. After evolving for some time, a new subpopulation, C, split from B, and again after some time a subpopulation D split from C. When all four populations evolve independently for some time after these splitting events and then are sampled for structure analysis, we may get *K* = 1, *K* = 2, *K* = 3, or *K* = 4, depending on the absolute and relative branch lengths of the phylogeny, and the sampling intensity of individuals and markers. For a given phylogeny, all 4 possible *K* values might be equally plausible. Indeed, a structure analysis at different *K* values (1,2,3,4) reveals differentiation patterns at different evolutionary scales and reveals different aspects of population structures. For example, 4 populations, A, B, C and D, might be inferred at *K* = 4, and 3 populations, A, B, and (C with D), might be inferred at *K* = 3, and so on. All inferences of the four possible *K* values are correct, apparently. Only when the inferences at *K* = 1, 2, 3, 4 are patched together do we get a complete picture of the population structure and evolutionary history. In the simple case of a star-like phylogeny or the island model assumed in most of my simulations, there does exist a single best *K*. However, it could still be difficult to infer *K* correctly when the populations are not much differentiated or are numerous, when markers are not much informative, or when samples from different populations are very small or highly unbalanced in size. For the ant data, all four estimators of *K* yielded a consistent result, *K* = 10, corresponding exactly to the sampled number of colonies. For the Arctic charr data, however, only 2 estimators (one for STRUCTURE and one for PopCluster) yield *K* = 6 which agrees with the number of sampling locations. The other 2 estimators yield *K* = 7 (Fig. 3). In practice, it is prudent to treat any unsupervised *K* inference from any estimator and admixture analysis with caution. When reliable external information (not used in structure analysis) such as sampling location is available, it is advised to make a final supervised determination of *K* by checking/comparing admixture inferences at different *K* values against external information and the *K* estimator.

Like any statistical model, the admixture model has many assumptions, no matter it is implemented in a Bayesian approach (e.g., STRUCTURE by Pritchard et al. 2000) or a likelihood approach (e.g., ADMIXTURE by Alexander et al. 2009; sNMF by Frichot et al. 2014; PopCluster of this study). One assumption is the independence of alleles both within and between loci. Independence of alleles within a locus of a diploid individual essentially assumes the absence of inbreeding (due to close relative mating), and the absence of admixture. While inbreeding causes a positive correlation, admixture leads to a negative correlation, between the paternal and maternal alleles of a diploid genotype. They produce too many and too few homozygotes, respectively, than those expected under Hardy-Weinberg equilibrium. However, violation of the assumption does not derail a model-based admixture analysis, as shown in this study (Supplementary Appendix 4). Even the extreme form of inbreeding, selfing, occurring at a high frequency (0.8) has almost negligible effects on admixture inference (Supplementary Fig. A4–4). Similarly, the methods can still recover the genetic structures accurately for highly admixed populations (Fig. 1C and Supplementary Fig. A4–1). The independence of alleles between loci (linkage equilibrium) is also violated often in practice. Linkage disequilibrium can occur even between unlinked loci due to factors such as non-random mating, genetic drift, selection and hybridisation. For linked loci, the disequilibrium is expected to be high because it dissipates slower over generations and thus could accumulate. However, except in the extreme case of all loci sampled from just one or a few small genomic regions (say, each region of 1 Mb in size), linkage disequilibrium should have rather limited effects on a model-based admixture analysis. Many admixture analyses on large genomic data, such as the human 1000 genome data with more than 38 million SNPs, yielded sensible results. A simulation study investigating the impact of linkage disequilibrium on admixture analysis is lacking, and future such studies should consider both model and non-model based admixture analysis methods on their robustness to linkage disequilibrium.

Using several empirical datasets and many simulated datasets in this study, I show PopCluster is in general advantageous over existing model-based admixture analysis methods. It is fast, capable of analysing both small multilocus genotype data such as a few microsatellites and large genomic data of millions of SNPs, and is accurate in various sampling conditions and actual population structures. However, in some situations, STRUCTURE could yield more robust and more accurate results than PopCluster. One of these situations is when sampling is highly unbalanced among populations and the markers are not highly informative (e.g., just a few microsatellites). In such a case, both PopCluster and STRUCTURE could recover the number of populations (as the optimal *K* value) represented by the sample, and reconstruct the admixture of the sample analysed at the optimal *K* value. For analyses conducted at a higher number of populations (say, *K* + 1 and *K* + 2), while STRUCTURE can still yield admixture estimates similar to those obtained at *K*, PopCluster sometimes splits the largest cluster into 2 or more clusters with “confidence” (i.e., with little admixture inferred for the individuals in the split clusters). Therefore, I suggest that, wherever possible, a real dataset be analysed by multiple admixture inference methods, with results carefully examined, compared among methods and with external information (such as sampling locations) not utilised in admixture analyses before reaching a conclusion.

The software package PopCluster described in this work is available for download from my website https://www.zsl.org/science/software/popcluster. It includes executables for Windows, Mac and Linux platforms, user’s guide and example datasets. For Windows, it also includes two additional components. One is a graphical user interface that facilitates the input of data and parameters, and the viewing of analysis results in tables and graphs (including the admixture stacked bar charts with user defined colours). The other is a simulation module that generates simulated genotype data under admixture, hybridisation or migration model for analysis by PopCluster, STRUCTURE or other methods.

## Supplementary information

Simulated annealing algorithm

EM algorithm for admixture analysis

Two estimators of *K*

Further simulations

Admixture analysis when samples are small

Admixture analysis when *K*=10 and *S*=20

Analysis of a simulated dataset of spatial admixture model

Admixture analysis with unbalanced sampling

Admixture analysis of a dataset with low differentiation

## Data Availability

The empirical datasets analysed and presented by this study are publicly available online. The simulation data are generated by the simulation module of the PopCluster software (Windows version) which is freely downloadable as stated in the manuscript.
