# Supplementary material for: Fast and accurate population admixture inference from genotype data from a few microsatellites to millions of SNPs
Source: Heredity (Edinb). 2022 May 4;129(2):79–92. doi: 10.1038/s41437-022-00535-z (PMC9338324; doi:10.1038/s41437-022-00535-z)
Supplement: Supplementary file 1 — Simulated annealing algorithm [file 41437_2022_535_MOESM1_ESM.pdf]

Supplementary Appendix 1: Simulated annealing algorithm

The simulated annealing algorithm proposed for a clustering analysis consists of the following steps.

1. *Generate an initial cluster configuration*. An initial configuration, $\mathbf{\Omega^{(0)}}$, is obtained by allocating the *N* sampled individuals at random into *K* nonempty clusters. Given $\mathbf{\Omega^{(0)}}$, initial allele frequencies, $\mathbf{P^{(0)}}$, are calculated by eqns (2-3). Given $\mathbf{\Omega^{(0)}}$ and $\mathbf{P^{(0)}}$, the initial log likelihood for each cluster and the total log likelihood of $\mathbf{\Omega^{(0)}}$ are calculated by (1) (or (5) with scaling) and (4), respectively.

2. *Generate a proposal configuration*. I use four types of proposals for modifying the current configuration to obtain a new configuration.

   2.1 *Move one individual*. One individual, i^* currently assigned to cluster k^*, is selected at random from the *N* individuals. The allele frequencies of cluster k^* are recalculated from (2-3) by removing individual i^*. The probability that i^* is reassigned to cluster *k* is calculated by

   $$\Pr{}'(k) = \prod_{l=1}^{L}\prod_{a=1}^{2} p_{klx_{i^*la}}, \quad \text{(A1)}$$

   where $p_{klx_{i^*la}}$ is the frequency of the *a*th allele at locus *l* of individual i^*, x_{i^*la} (=1, 2, …, J_l), in population *k*, for *k*=1,2,…,*K*. A smaller cluster with fewer individual members would be disfavoured for assignment because genetically it is more likely to differ from individual i^*. In the extreme case of a cluster containing only 1 individual, for example, many alleles in i^* may be absent from the cluster. To encourage assignments to small clusters, the probability of assignment to a cluster *k* is scaled by its relative size, N_k/N, and then normalized

   $$\Pr(k) = (\Pr{}'(k))^{N_k/N} / \sum_{m=1}^{K}(\Pr{}'(m))^{N_m/N}. \quad \text{(A2)}$$

   Individual $i*$ is assigned to cluster k' according to probability distribution (A2). If $k' \neq k^*$, then it is a new configuration whose likelihood will be calculated to determine whether it is to be accepted or rejected (below). Otherwise, the proposal changes nothing and is regarded as a failure and is rejected.

   2.2 *Swap two individuals*. Follow the same procedure as in 2.1 to determine i^*, k^* and k'. The proposal is rejected, and the next iteration is initiated if $k' = k^*$. Otherwise, I choose one individual, i', currently in cluster k' to swap its cluster membership with i^*. For each individual in k', I calculate reassignment probability $\Pr(k^*)$ by (A2). The individual with the maximum value of $\Pr(k^*)$ is chosen as i'. The new configuration is obtained by assigning individual i^* to cluster k' and individual i' to cluster k^*.

   2.3 *Merge two clusters*. Calculate $F_{IS(k)}$ (i.e. inbreeding within cluster *k*) of each cluster *k*, and choose the two clusters, say $k'$and k^*, with the smallest $F_{IS(k)}$ values

to merge into a single cluster, k'. If the new configuration is accepted, there will be one empty cluster, k^*.

2.4 *Split one cluster into two clusters*. When there exists one empty cluster, I choose the cluster that has the highest $F_{IS(k)}$ value and has 2 or more individual members to split it into two nonempty clusters by selecting individuals at random. If the new configuration is accepted, there will be K nonempty clusters.

3. *Accept or reject the new configuration*. Calculate the log likelihood of the new configuration, $\mathcal{L}(\boldsymbol{\Omega}')$, by (4) and compare it with the log likelihood of the old configuration, $\mathcal{L}(\boldsymbol{\Omega})$, to determine whether to accept or reject $\boldsymbol{\Omega}'$ by Metropolis algorithm. Calculate $\tau = \text{Min}(e^{(\mathcal{L}(\boldsymbol{\Omega}')-\mathcal{L}(\boldsymbol{\Omega}))/T}, 1)$, where T is the annealing temperature governing the rate at which a new configuration is accepted. Generate a random number, r, uniformly distributed between 0 and 1. The proposal is regarded successful and $\boldsymbol{\Omega}'$ is thus accepted when $r \leq \tau$; otherwise, $\boldsymbol{\Omega}'$ is rejected and $\boldsymbol{\Omega}$ is recovered. Note in calculating $\mathcal{L}(\boldsymbol{\Omega}')$, the likelihood of only one (for a proposal by procedure 2.3) or two (for a proposal by other procedures) clusters need to be recalculated. This clustering algorithm is thus computationally efficient, especially for a large scale clustering analysis with many populations.

4. *Repeat steps 2–3 by a sufficiently large number of times*. Among the 4 types of proposals, 2.4 is adopted only when there exist empty clusters, 2.3 is adopted at a very low frequency, 2.1 and 2.2 are adopted at frequencies proportional to $1 - K/N$ and K/N, respectively. The most frequently adopted proposal is from 2.1. However, other types of proposals are also necessary, especially when clusters (each having on average N/K individuals) are small, to reduce the risk of converging to a local maximum. This iterative procedure ensures the likelihood to increase (i.e. to climb uphill) in general, but also allows the likelihood to decrease (i.e. to descend downhill for traversing a valley) occasionally to avoid the algorithm getting stuck on a local maximum. The probability of a downhill tour is controlled by T, which is decreased as the annealing process proceeds so that an inferior proposal becomes less and less frequently accepted. Initially T is set a value to allow roughly a 60% acceptance rate of new configurations. It is then reduced in multiplicative steps, each amounting to a 10% decrease. A given T value is held in $100NK$ reconfigurations or in $10NK$ successfully accepted reconfigurations, whichever comes first. When efforts to improve configurations become sufficiently discouraging (i.e. acceptance rate is close to zero), the iterative process is stopped and the best configuration with the maximum likelihood is taken as the best estimate.
