# Supplementary material for: Fast and accurate population admixture inference from genotype data from a few microsatellites to millions of SNPs
Source: Heredity (Edinb). 2022 May 4;129(2):79–92. doi: 10.1038/s41437-022-00535-z (PMC9338324; doi:10.1038/s41437-022-00535-z)
Supplement: Supplementary file 2 — EM algorithm for admixture analysis [file 41437_2022_535_MOESM2_ESM.pdf]

Supplementary Appendix 2: EM algorithm for admixture analysis

To facilitate the use of an EM algorithm, I follow previous studies (e.g. Pritchard et al. 2000; Tang et al. 2005) by introducing an auxiliary variable $z_{ila} \in \{1,2, \dots, K\}$, which designates the source population of allele a (=1, 2) at locus l (=1, 2, …, L) in individual i (=1, 2, …, N). By using z_{ila}, the augmented log likelihood function (6) becomes

$$\mathcal{L}(\mathbf{Q}, \mathbf{P}|\mathbf{X}, \mathbf{Z}) = \sum_{i=1}^{N} \sum_{l=1}^{L} \sum_{a=1}^{2} \mathrm{Log}\left(q_{iz_{ila}} p_{z_{ila} l x_{ila}}\right). \quad \text{(A2-1)}$$

Parameters **Q** and **P** could be calculated straightforwardly from **Z**, if it were known. However, **Z** is unobservable. It (its expectation) can be inferred from **X** and then used in calculating **Q** and **P**.

Initial values of **Q** *and* **P**: Suppose an individual i is assigned to cluster k in the clustering analysis. Its initial admixture proportions are then set as $q_{im}^{(0)} = d - 1/(K+2)$ if m=k, and $q_{im}^{(0)} = (1 - q_{ik})/(K-1)$ if $m \neq k$. The value of d determines the extent we believe in the mixture model and the validity of clustering analysis results. A wide range of d values, from 0.5 to 1.0, produced similarly good results in many testing analyses of simulated data. In this study I choose a value of d=0.9 in all analyses of simulated and empirical data. Given initial values $\mathbf{Q}^{(0)}$, the initial count of copies of allele j at locus l in cluster k is calculated by

$$c_{klj}^{(0)} = \sum_{i=1}^{N} \sum_{a=1}^{2} \delta_{jx_{ila}} q_{ik}^{(0)}, \quad \text{(A2-2)}$$

where the Kronecker delta $\delta_{jx_{ila}} = 1$ and 0 if $j = x_{ila}$ and $j \neq x_{ila}$, respectively. Given these $c_{klj}^{(0)}$ values, the initial allele frequencies $p_{klj}^{(0)}$ are calculated by (2) and (3).

Iterations to update **Q**: Given allele frequencies $\mathbf{P}^{(0)}$, an individual i's admixture proportions are updated iteratively from the initial values $\mathbf{q}_i^{(0)} = \{q_{i1}^{(0)}, q_{i2}^{(0)}, \dots, q_{iK}^{(0)}\}$. Allele frequencies at each locus l in each population k are calculated by excluding the alleles in individual i from allele count $c_{klj}^{(0)}$ to obtain $\mathbf{P}^{(i0)}$. Given $\mathbf{q}_i^{(0)}$ and $\mathbf{P}^{(i0)}$, the expectation of the auxiliary variable z_{ila} (a=1, 2) being k is computed by Bayes rule

$$E_{ilak}^{(1)} = \frac{p_{klx_{ila}}^{(i0)} q_{ik}^{(0)}}{\sum_{m=1}^{K} p_{mlx_{ila}}^{(i0)} q_{im}^{(0)}}. \quad \text{(A2-3)}$$

q_{ik} is then updated by using $E_{ilak}^{(1)}$,

$$q_{ik}^{(1)} = \frac{\sum_{l=1}^{L} \sum_{a=1}^{2} E_{ilak}^{(1)}}{\sum_{l=1}^{L} \sum_{a=1}^{2} \sum_{m=1}^{K} E_{ilam}^{(1)}}. \quad \text{(A2-4)}$$

Eqns (A2-3) and (A2-4) are used to update $\mathbf{q}_i^{(n)}$ from $\mathbf{q}_i^{(n-1)}$ via $E_{ilak}^{(n)}$ until quasi convergence, with n=1, 2, 3, ...

Iterations to update **P**: Corresponding to the converged $\mathbf{q}_i$ values, $\boldsymbol{q}_i^* = \{q_{i1}^*, q_{i2}^*, \dots, q_{iK}^*\}$ for *i*=1, 2, …, *N*, the E_{ilak}^* values are used to update **P**,

$$p_{klj}^{(1)} = \frac{\sum_{i=1}^{N}\sum_{a=1}^{2} E_{ilak}^{*}\delta_{jx_{ila}}}{\sum_{i=1}^{N}\sum_{a=1}^{2} E_{ilak}^{*}}. \quad \text{(A2-5)}$$

The above iterations for updating **Q** and **P** via **E** are repeated until both **Q** and **P** are asymptotically converged. At iteration *n*, I calculate the change of **Q** by $t_n = \frac{1}{NK}\sum_{i=1}^{N}\sum_{k=1}^{K}\left(q_{ik}^{(n)} - q_{ik}^{(n-1)}\right)^2$ and compare it to a convergence tolerance threshold value τ. Convergence is deemed reached when $t_n < \tau$, where τ is a small value such as 10^{-6}. The final converged **Q** and **P** are the admixture estimates and allele frequency estimates, respectively, under the assumed admixture model.
