# Supplementary material for: Fast and accurate population admixture inference from genotype data from a few microsatellites to millions of SNPs
Source: Heredity (Edinb). 2022 May 4;129(2):79–92. doi: 10.1038/s41437-022-00535-z (PMC9338324; doi:10.1038/s41437-022-00535-z)
Supplement: Supplementary file 3 — Two estimators of K [file 41437_2022_535_MOESM3_ESM.pdf]

Supplementary Appendix 3: Two estimators of *K*

1. Estimator D_{LK2}

The computational procedure of D_{LK2} is as follows. For each value k of the number of source populations in the predetermined range [k_0, k_1], n replicate clustering analyses are conducted using different random number seeds. Suppose the maximum log-likelihood among the n replicate runs at a given k is $\mathcal{L}_{k(0)}$. The first order rate of change of log-likelihood at k is $D_{k,1} = \frac{\mathcal{L}_{k(0)} - \mathcal{L}_{k-1(0)}}{(|\mathcal{L}_{k(0)}|+|\mathcal{L}_{k-1(0)}|)/2}$, for $k = k_0 + 1, k_0 + 2, \ldots, k_1$. The second order rate of change at k is $D_{Lk2} = \frac{D_{k,1} - D_{k+1,1}}{(|D_{k,1}|+|D_{k+1,1}|)/2}$, for $k = k_0 + 1, k_0 + 2, \ldots, k_1 - 1$. The value of k which gives the maximal value of D_{Lk2} is the estimate of K, the most likely number of source populations represented by the sampled individuals.

2. *Estimator F_{STIS}*

The computational procedure of F_{STIS} is as follows. For each k value in the predetermined range [k_0, k_1], n replicate clustering analyses are conducted using different random number seeds. The strength of the identified structure at k is measured by $S_k = \left(\sum_{i=1}^{n}\sum_{m=1}^{k} F_{ST,im}\right) / \left(\sum_{i=1}^{n}\sum_{m=1}^{k} |F_{IS,im}|\right)$, where $F_{ST,im}$ and $F_{IS,im}$ are the estimated F_{ST} and F_{IS} values, respectively, for the mth (=1,2,…,k) cluster obtained in the ith (=1,2,…,n) replicate run. $F_{ST,im}$ and $F_{IS,im}$ are estimated from the genotype data for a given clustering configuration, using the method of Nei and Chesser (1983). The absolute F_{IS} values are used in the calculation because the magnitude of deviation from equilibrium counts, and the direction of deviation (positive or negative) is irrelevant. The value of k which gives the maximal value of S_k acts as the estimate of K.

Note neither D_{LK2} nor F_{STIS} applies when the true K is 1, a single source population. This is because, when k=1, D_{LK2} becomes undefined and $F_{STIS} \equiv 0$. To determine whether K=1 or K>1, we may have to rely on the likelihood (or related statistics) of the models. In analysing the simulated and empirical data (below), I found D_{LK2} is usually more accurate than F_{STIS}, and therefore estimates of K presented in this study were obtained by using D_{LK2} only.

References

Nei M, Chesser R (1983) Estimation of fixation indices and gene diversities. Ann Hum Genet 47:253–259.
