# Supplementary material for: Fast and accurate population admixture inference from genotype data from a few microsatellites to millions of SNPs
Source: Heredity (Edinb). 2022 May 4;129(2):79–92. doi: 10.1038/s41437-022-00535-z (PMC9338324; doi:10.1038/s41437-022-00535-z)
Supplement: Supplementary file 4 — Further simulations [file 41437_2022_535_MOESM4_ESM.pdf]

## Supplementary Appendix 4: Further simulations

*Simulation A1, high admixture*

The presence of many highly admixed individuals in a sample poses challenges to a population structure analysis. These individuals may blur the boundaries of source populations and make it difficult to estimate both **P** and **Q**. The admixture extent of an individual *i* can be measured by the probability that two alleles drawn at random from it come from different source populations, $M_i = 1 - \sum_{k=1}^{K} q_{ik}^2$. The admixture extent of a sample of *N* individuals can be measured by its average individual admixture, $\bar{M} = \sum_{i=1}^{N} M_i / N$. I use an admixture probability, *d*, to regulate $\bar{M}$ in simulations. For an individual *i*, a random number, $r_1$, is drawn from the uniform distribution and compared with *d*. The individual is determined to be admixed and purebred when $r_1 < d$ and otherwise, respectively. In the former case, I assume the number of source populations contributing to the admixed individual, $n_d$, follows a triangular distribution, $\Pr(n_d) \sim 1/(n_d - 1)$ for $n_d = 2,3,\dots,K$. The actual number of contributing source populations, $n_{di}$, is determined by drawing another uniformly distributed random number, $r_2$, and comparing it to the triangular distribution (probabilities normalized to sum to 1 over $n_d = 2,3,\dots,K$). Given $2 \le n_{di} \le K$, a number of $n_{di}$ uniformly distributed random numbers are then generated and normalized (such that they sum to 1) as the ancestry coefficients (i.e. true $\mathbf{q}_i$) of individual *i*. The genotype of individual *i* at each locus is then generated independently given the simulated $\mathbf{q}_i$, and the allele frequencies of the source populations.

The simulation results for a varying value of *d* are shown in Figure A4-1.

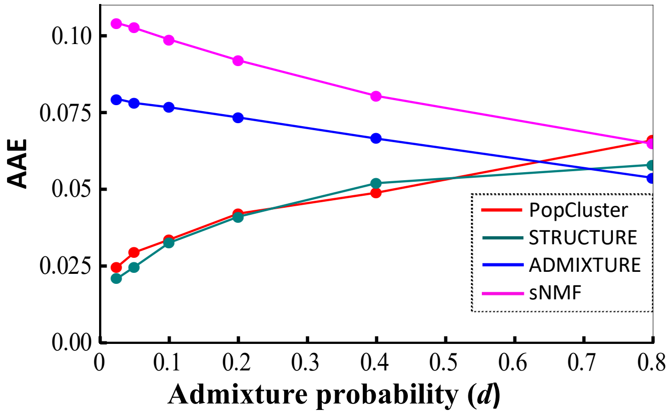


**Fig. A4-1 Average assignment error, AAE, as a function of admixture probability (*d*).** *K*=3 populations with $F_{ST}$=0.1 in the island model were simulated, 50 individuals were sampled from each population, and each sampled individual was genotyped at 1000 SNP loci.

*Simulation A2, relatedness*

Bayesian and likelihood admixture models assume that individuals are sampled at random from a large outbred population. They essentially assume, in part, that individuals from each subpopulation are unrelated (with respect to the source sampling subpopulation), having no substructure such as family structure. In practice, the assumption can be violated in some sampling schemes for fecund species. For example, localized sampling of individuals in early-life stages of a fecund species (e.g. eggs or tadpoles of frogs) may yield a sample containing large family structures. The presence of family structures in a sample may confuse admixture analysis methods and interfere with population structure inference. As a result, it could lead to the misidentification of a family as a subpopulation, and the overestimation of the number of subpopulations (Guinand et al. 2006; Anderson and Dunham 2008; Rodríguez-Ramilo and Wang 2012). No study has been conducted to investigate whether admixture analysis methods other than STRUCTURE suffer from the same sampling problem or not. In the present work, I simulate different proportions of sampled individuals from a subpopulation as full siblings, assuming a Poisson distribution of the size of a full-sib family included in a subsample taken from a subpopulation (Table 1). Different methods were applied to the data analysis by assuming the simulated number of subpopulations $K$, to compare the robustness and accuracy of the methods to the presence of familial relatedness.

The simulation results are shown in Figure A4-2.

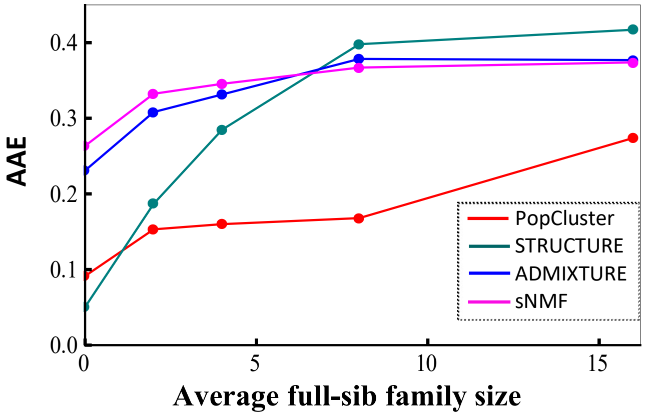


**Fig. A4-2 Average assignment error, AAE, as a function of average full-sib family size (*x* axis).** The island model of a number of $K$=3 populations with $F_{ST}$ = 0.025 was simulated. A subsample of 50 individuals was sampled from each population, and each individual was genotyped at a number of $L = 1000$ SNP loci.

For one of the replicate datasets simulated with an average full-sib family size of 4, the estimated pairwise relatedness, the simulated admixture, and the estimated admixture by STRUCTURE, PopCluster, ADMIXTURE and sNMF are shown in Figure A4-3. As can be seen, the presence of family structure causes the inference of sporadic admixture, especially by programs ADMIXTURE and sNMF.

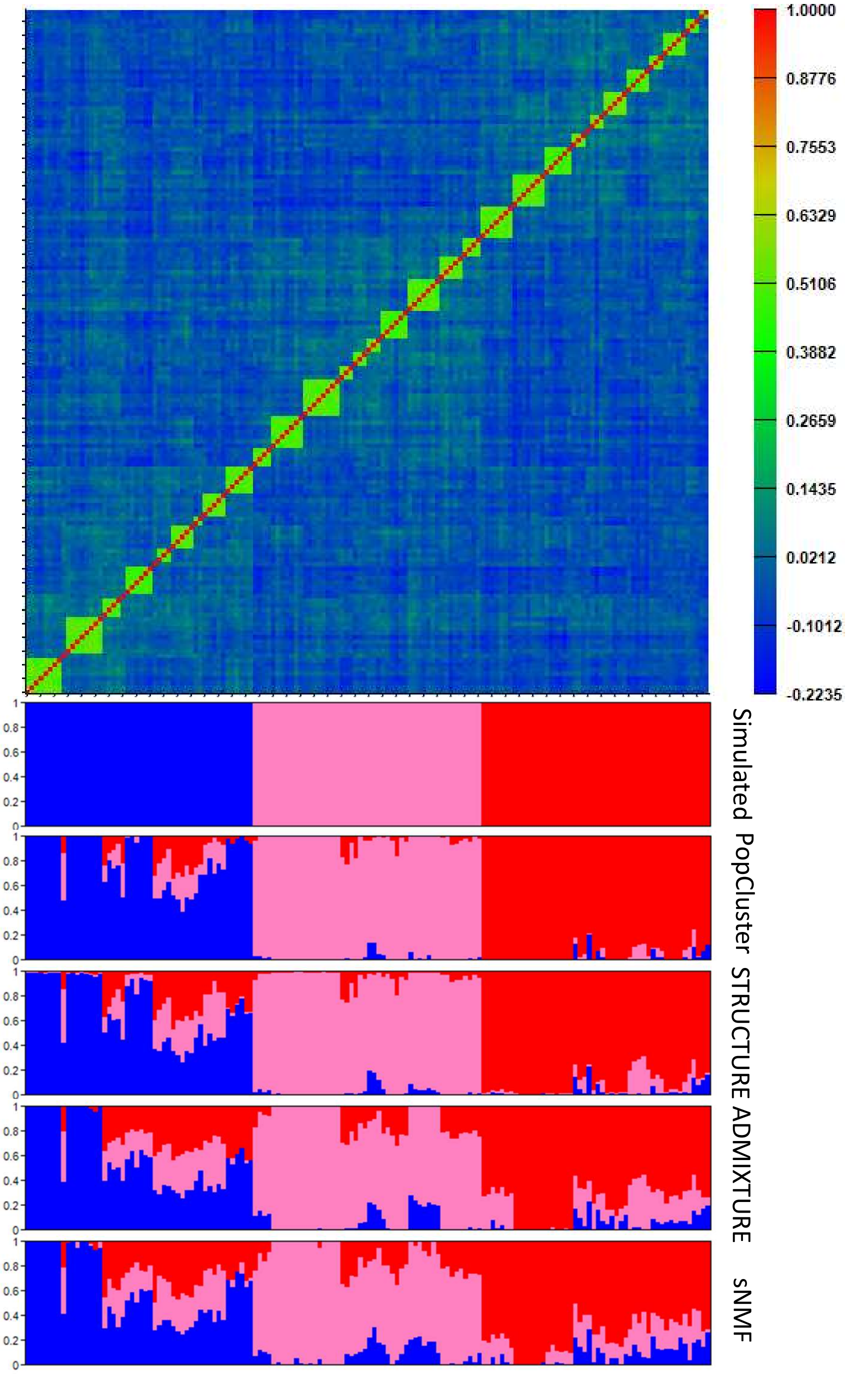


**Fig. A4-3 Simulated and estimated admixture and estimated pairwise relatedness for a dataset simulated with an average full-sib family size of 4.** The island model of $K$=3 populations with $F_{ST}$ = 0.025 was simulated. A subsample of 50 individuals was sampled from each population, and each individual was genotyped at a number of $L = 1000$ SNP loci. Individuals 1, 2, …, 150 are on both $x$ and $y$ axes of the heatmap (plotted by PopCluster) for pairwise relatedness.

*Simulation A4-3, inbreeding*: Almost all (but see Gao et al. 2007) model-based methods for population structure analysis assume independence of an individual's paternal and maternal alleles at a locus. The assumption is violated in the presence of admixture or inbreeding which result in too few or too many homozygotes, respectively, than those expected under Hardy-Weinberg equilibrium. STRUCTURE and other methods are robust to the presence of admixture, as demonstrated by analysing simulated and empirical datasets (Pritchard et al. 2000). Otherwise, such methods would have much limited use in practice. Gao et al. (2007) showed that STRUCTURE performs poorly in the presence of selfing. No study has been conducted to confirm or refute the conclusion, and to check the performance of other methods in analysing data with inbreeding. In this study, I simulated data with varying selfing rates (Table 1) and analysed the data by different methods to compare their robustness to inbreeding.

The simulation results are shown in Figure A4-4.

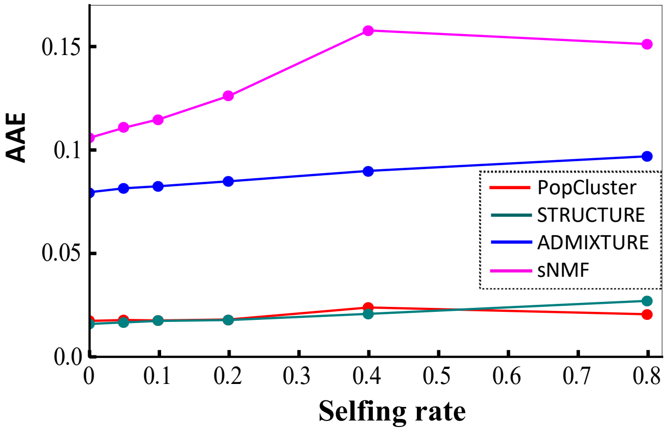


**Fig. A4-4 Average assignment error, AAE, as a function of selfing rate**. The island model of $K$=3 populations with $F_{ST}$ = 0.1 was simulated. A subsample of 50 individuals was sampled from each population, and each individual was genotyped at $L$ = 1000 SNP loci.

For a particular dataset simulated with a selfing rate of 0.8, the simulated and estimated admixture is shown in Figure A4-5. Both ADMIXTURE and sNMF overestimate admixture substantially.

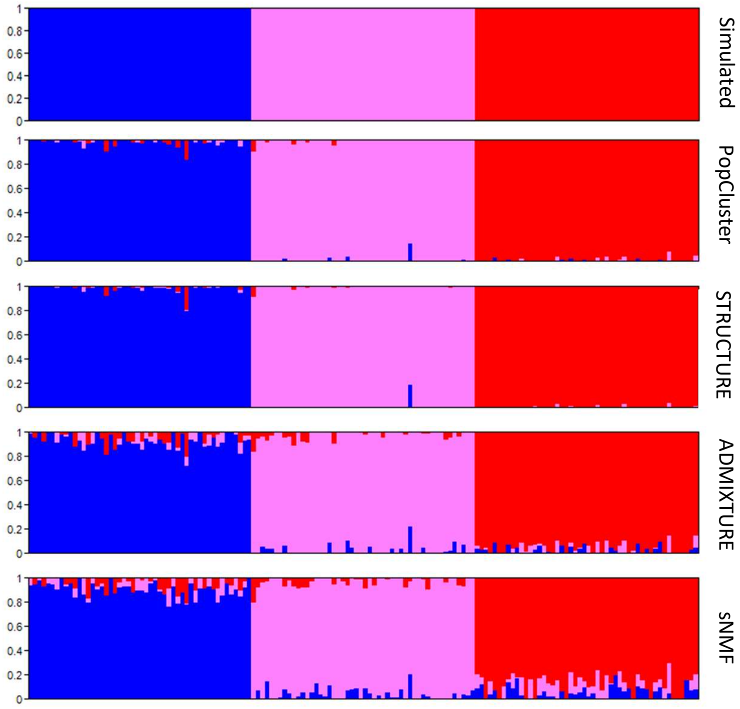


**Fig. A4-5 Simulated and estimated admixture for a dataset simulated with a selfing rate of 0.8.** The island model of $K$=3 populations with $F_{ST}$ = 0.1 was simulated. A subsample of 50 individuals was sampled from each population, and each individual was genotyped at $L$ = 1000 SNP loci.

## References

Anderson EC, Dunham KK (2008) The influence of family groups on inferences made with the program STRUCTURE. Mol Ecol Res 8:1219–1229.

Gao H, Williamson S, Bustamante CD (2007) A Markov chain Monte Carlo approach for joint inference of population structure and inbreeding rates from multilocus genotype data. Genetics 176:1635-1651.

Guinand B, Scribner KT, Page KS, Filcek K, Main L, Burnham-Curtis MK (2006) Effects of coancestry on accuracy of individual assignments to populations of origin: examples using Great Lakes lake trout (*Salvelinus namaycush*). Genetica 127:329–340.

Rodríguez-Ramilo ST, Wang J (2012) The effect of close relatives on unsupervised Bayesian clustering algorithms in population genetic structure analysis. Mol Ecol Res 12:873-884.
