# Supplementary material for: Fast and accurate population admixture inference from genotype data from a few microsatellites to millions of SNPs
Source: Heredity (Edinb). 2022 May 4;129(2):79–92. doi: 10.1038/s41437-022-00535-z (PMC9338324; doi:10.1038/s41437-022-00535-z)
Supplement: Supplementary file 5 — Admixture analysis when samples are small [file 41437_2022_535_MOESM5_ESM.pdf]

Supplementary Appendix 5: Admixture analysis when samples are small

Figure 1A summarises and compares the accuracy of the 4 admixture analysis methods when the subsample from each of the 10 source populations is small on average. It is interesting to find out how and why the 4 methods differ in accuracy as shown in Figure 1A, revealed by examining the individual admixture estimates. Figure A5-1 shows the simulated and inferred individual admixture of a particular simulated dataset with a subsample size of 4 individuals from each of the 10 source populations. The other parameters used in simulating the data are the same as those of Figure 1A.

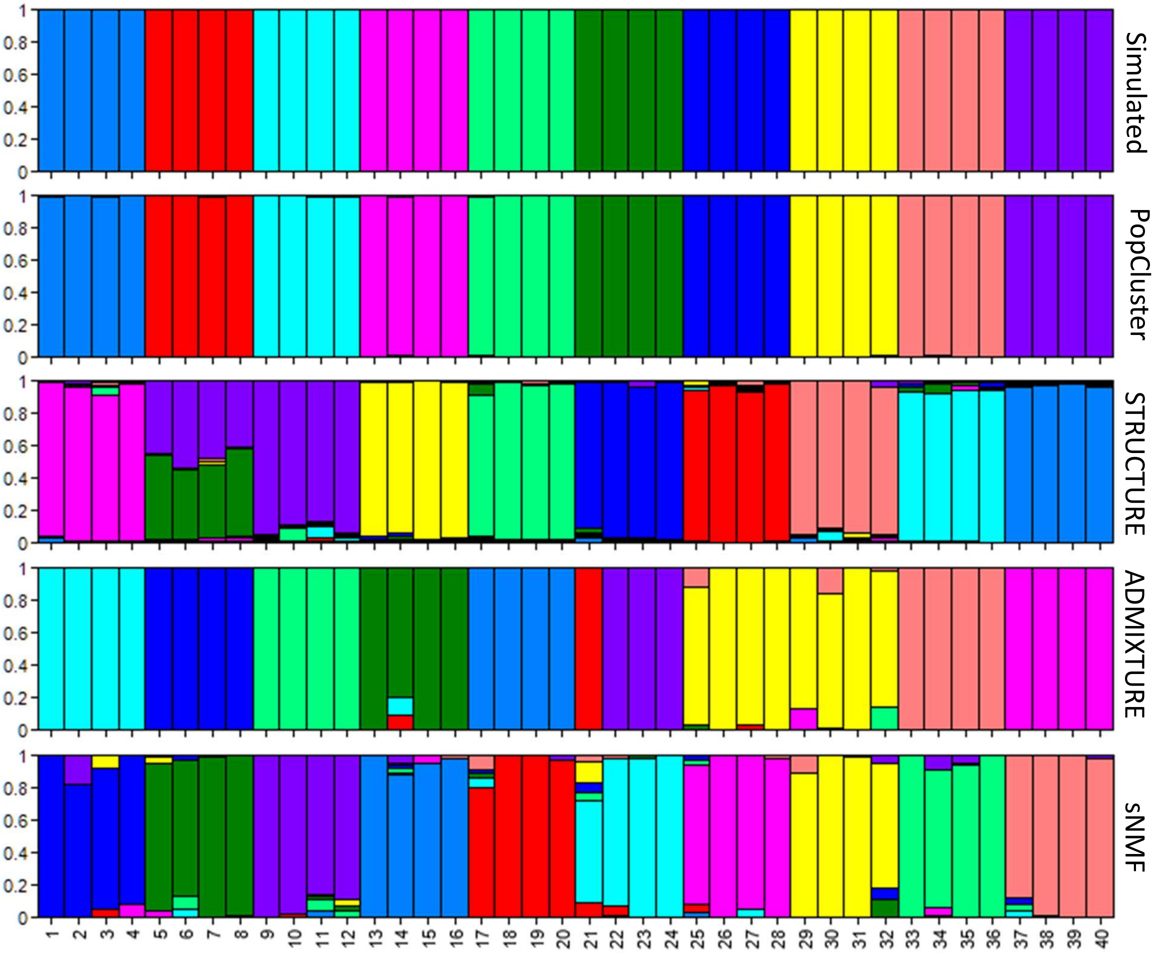

Fig. A5-1 Simulated and estimated individual admixture of a simulated dataset. Four individuals are sampled from each of 10 source populations simulated with F_{ST}=0.1 in the island model. Each sampled individual was genotyped at 1000 SNP loci.
