# Supplementary material for: Fast and accurate population admixture inference from genotype data from a few microsatellites to millions of SNPs
Source: Heredity (Edinb). 2022 May 4;129(2):79–92. doi: 10.1038/s41437-022-00535-z (PMC9338324; doi:10.1038/s41437-022-00535-z)
Supplement: Supplementary file 6 — Admixture analysis when K=10 and S=20 [file 41437_2022_535_MOESM6_ESM.pdf]

Supplementary Appendix 6: Admixture analysis when *K*=10 and *S*=20

Figure 1B summarises and compares the accuracy of the 4 admixture analysis methods when many source populations are sampled, with each population being represented by 20 individuals. The inaccuracy of the 4 methods as shown in Figure 1B is caused by their false admixture inferences. The individual admixture simulated and inferred by each method for a particular simulated dataset is shown in Figure A6-1.

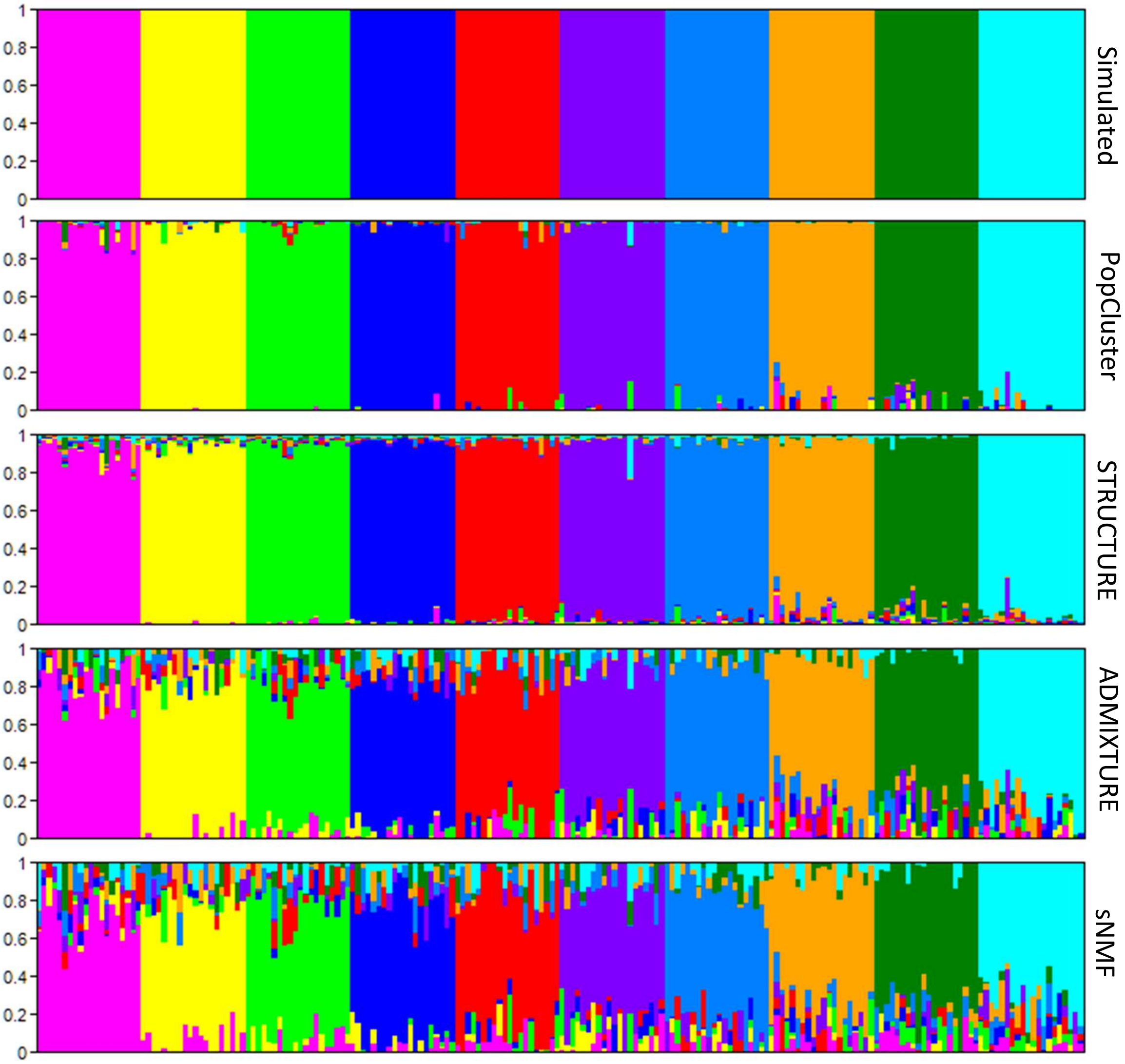

Fig. A6-1 Simulated and estimated individual admixture of a simulated dataset. Twenty individuals are sampled from each of 10 source populations simulated with F_{ST}=0.05 in the island model. Each sampled individual was genotyped at 1000 SNP loci.
