# Supplementary material for: Fast and accurate population admixture inference from genotype data from a few microsatellites to millions of SNPs
Source: Heredity (Edinb). 2022 May 4;129(2):79–92. doi: 10.1038/s41437-022-00535-z (PMC9338324; doi:10.1038/s41437-022-00535-z)
Supplement: Supplementary file 7 — Analysis of a simulated dataset of spatial admixture model [file 41437_2022_535_MOESM7_ESM.pdf]

Supplementary Appendix 7: Analysis of simulated dataset of spatial admixture model

Figure 1C summarises and compares the accuracy of the 4 admixture analysis methods applied to the analyses of data simulated under the spatial admixture model. For a particular dataset simulated with $Q_{11} = 0.9$, the simulated admixture of the 500 individuals (100 from each of the 5 populations) and the estimated admixture from the 4 methods are shown in Figure A7-1.

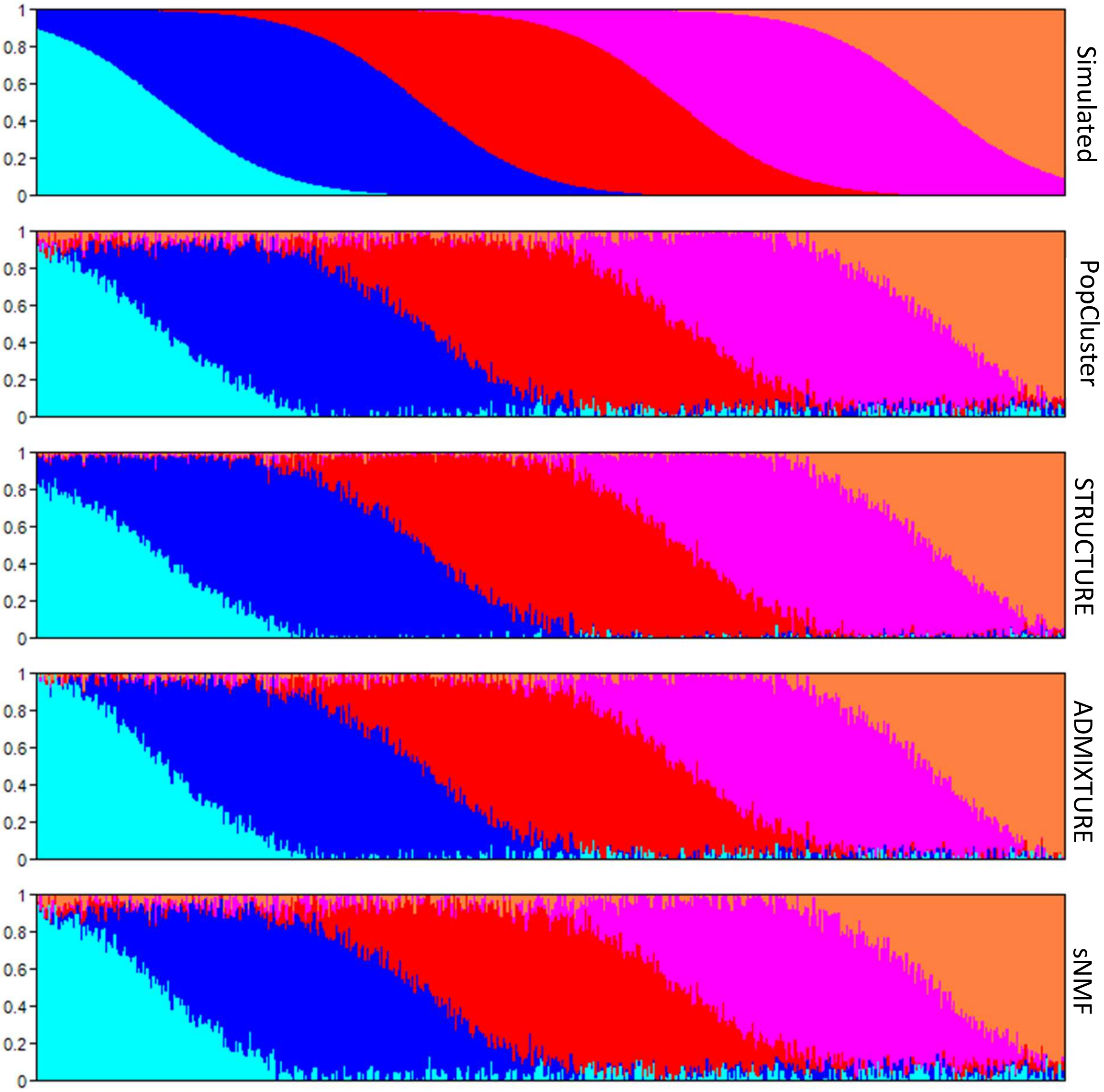

Fig. A7-1 Simulated and estimated individual admixture of a dataset simulated under the spatial admixture model. One hundred individuals are sampled from each of 5 source populations simulated with q_{11} =0.9 in the spatial admixture model. Each sampled individual was genotyped at 10000 SNP loci.
