# Supplementary material for: Fast and accurate population admixture inference from genotype data from a few microsatellites to millions of SNPs
Source: Heredity (Edinb). 2022 May 4;129(2):79–92. doi: 10.1038/s41437-022-00535-z (PMC9338324; doi:10.1038/s41437-022-00535-z)
Supplement: Supplementary file 8 — Admixture analysis with unbalanced sampling [file 41437_2022_535_MOESM8_ESM.pdf]

Supplementary Appendix 8: Admixture analysis with unbalanced sampling

Figure 1E summarises and compares the accuracy of the 4 admixture analysis methods when sampling is unbalanced to a varying degree among the 3 source populations. For a particular one of the datasets simulated with sample sizes of 276, 12 and 12 for populations 1, 2 and 3 respectively, a number of 1000 or 100 SNPs, and other parameters as shown in Figure 1E, the actual (simulated) and inferred individual admixture by the 4 analysis methods is shown in Figure A8-1.

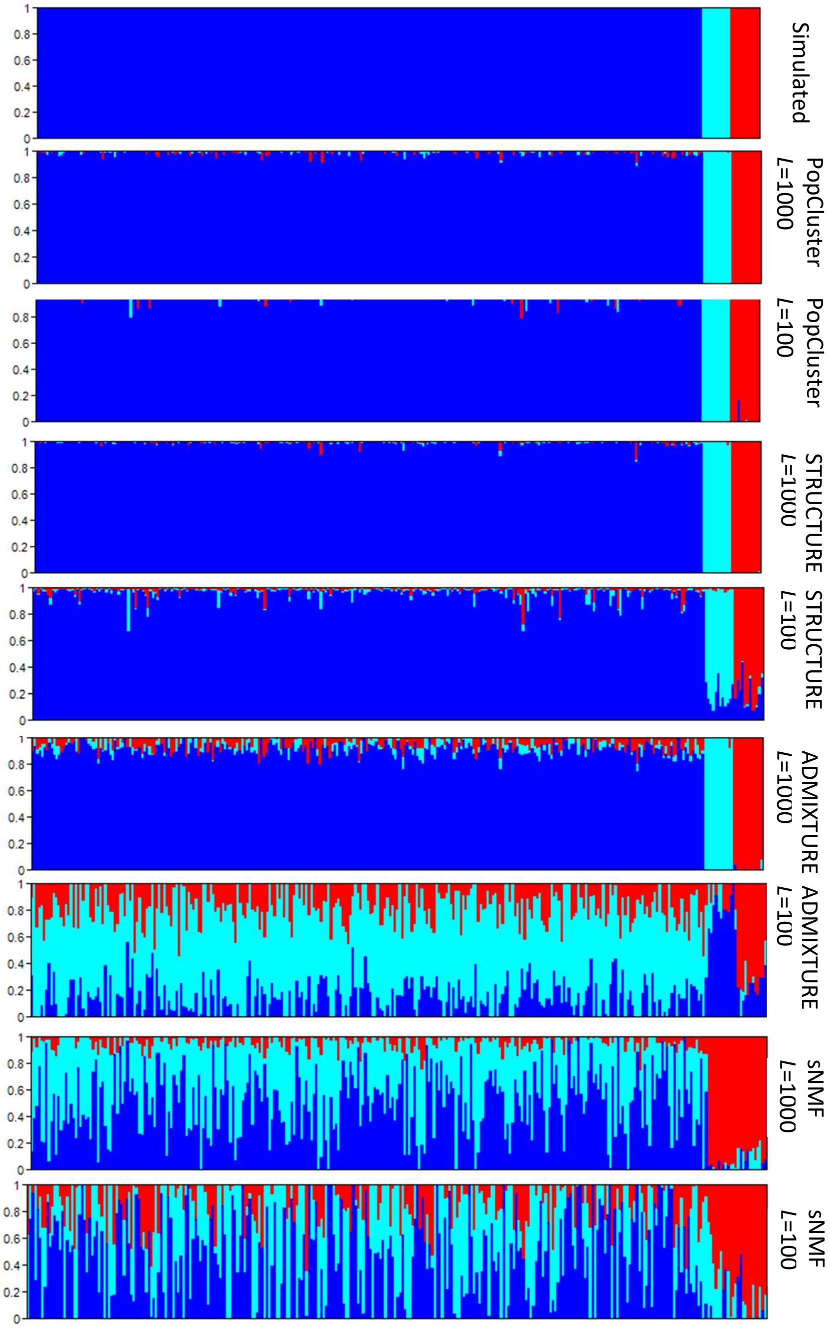

Fig. A8-1 Simulated and estimated individual admixture of a simulated dataset. 176, 12 and 12 individuals are sampled from 3 source populations simulated with F_{ST}=0.1 in the island model. Each sampled individual was genotyped at 1000 SNP loci. Admixture analysis was conducted using either all of the 1000 loci or just the first 100 loci.
