# Supplementary material for: Fast and accurate population admixture inference from genotype data from a few microsatellites to millions of SNPs
Source: Heredity (Edinb). 2022 May 4;129(2):79–92. doi: 10.1038/s41437-022-00535-z (PMC9338324; doi:10.1038/s41437-022-00535-z)
Supplement: Supplementary file 9 — Admixture analysis of a dataset with low differentiation [file 41437_2022_535_MOESM9_ESM.pdf]

Supplementary Appendix 9: Admixture analysis of a dataset with low differentiation

Figure 1D summarises and compares the accuracy of the 3 admixture analysis methods when populations have low levels of differentiation. For a particular one of the datasets simulated with parameters F_{ST}= 0.001, L=1000000, sample size of 50 individuals for each of the three source populations, two alleles per locus, the actual (simulated) and inferred individual admixture by the 3 analysis methods is shown in Figure A9-1. Two replicate runs were conducted on this dataset by each of the three methods, using the same parameter setting except for random number seeds. The two replicate runs of PopCluster yield identical results for both maximum loglikelihood value, which is -1.4989072493E+08, and admixture estimates (Figure A9-1). The two replicate runs of sNMF have different least-square errors, which are 68556237.6 and 68555652.7, and have slightly different admixture estimates (Figure A9-1) with the first run showing higher admixture than the second run. The two replicate ADMIXTURE runs have maximum loglikelihood values -148382592.7 and -148383124.4, and have slightly different admixture estimates (Figure A9-1). Both sNMF and ADMIXTURE overestimate admixture, especially sNMF.

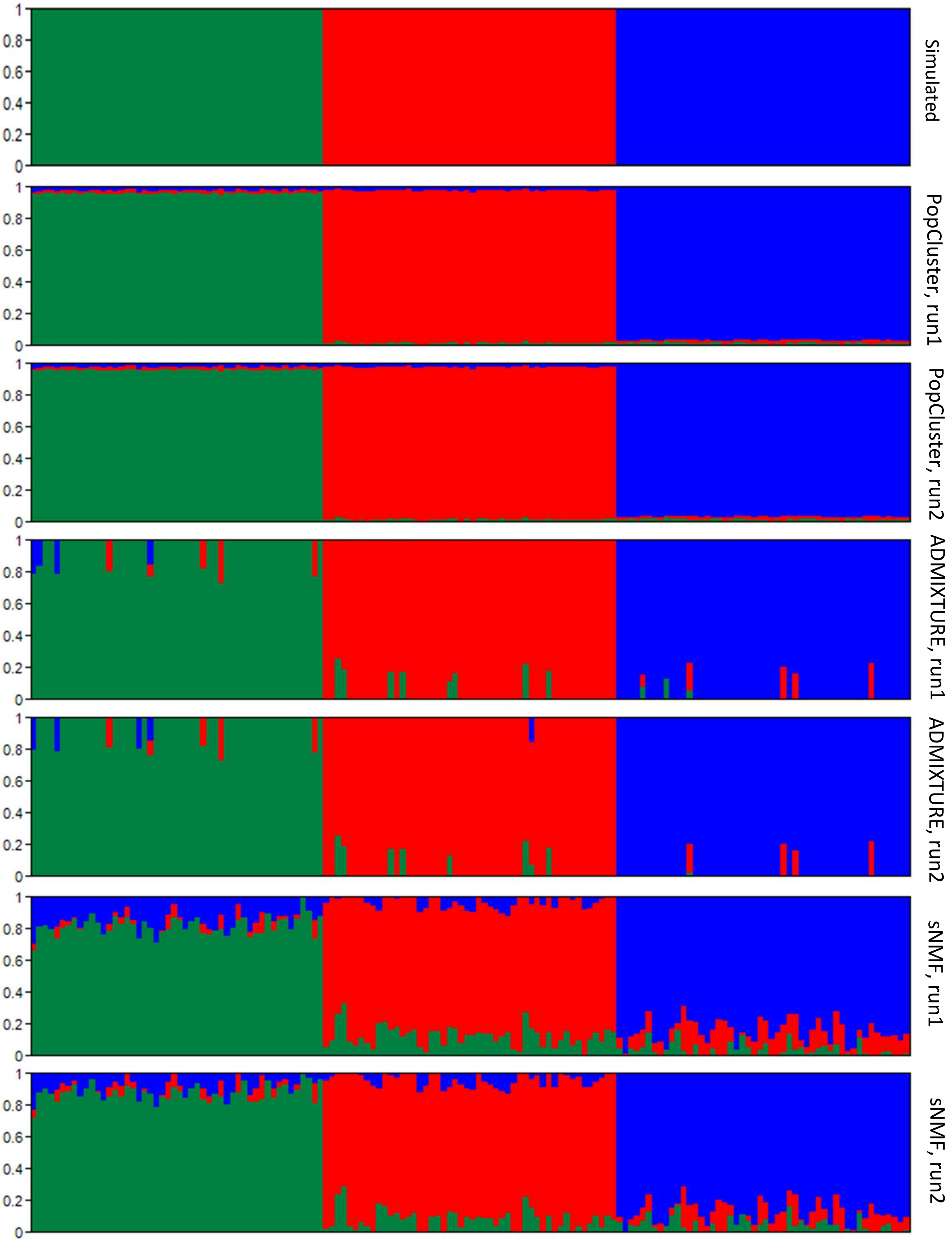

Fig. A9-1 Simulated and estimated individual admixture of a simulated dataset with F_{ST} = 0.001. Other parameters are the same as in Figure 1D and listed in Table 1. Two replicate runs were conducted using PopCluster, ADMIXTURE and sNMF.
